# Design, Synthesis, and Biological Evaluation of Benzimidazol‐2‐One Hybrids as Potential Anti‐Alzheimer's Disease Agents

**DOI:** 10.1002/ddr.70369

**Published:** 2026-08-20

**Authors:** Fatih Yılmaz, Emre Menteşe, Ozan Emre Eyüpoğlu, Bahittin Kahveci, Mustafa Emirik

**Affiliations:** ^1^ Department of Chemistry and Chemical Process Technology, Vocational School of Technical Sciences Recep Tayyip Erdogan University Rize Turkey; ^2^ Department of Chemistry, Faculty of Art and Sciences Recep Tayyip Erdogan University Rize Turkey; ^3^ Department of Biochemistry, School of Pharmacy Istanbul Medipol University Istanbul Turkey; ^4^ Department of Nutrition and Dietetics, Faculty of Health Sciences Karadeniz Technical University Trabzon Turkey

**Keywords:** AChE, Alzheimer's disease, BChE, benzimidazolone, coumarin, MAO‐B, online HPLC analysis, triazole

## Abstract

Alzheimer's disease (AD) is a multifactorial neurodegenerative disorder for which single‐target therapies often provide insufficient benefit, motivating the development of multi‐target‐directed ligands (MTDLs). In this study, a novel series of benzimidazolone‐based hybrids incorporating piperazine, coumarin, and triazole moieties was designed, synthesized, and evaluated for inhibitory activity against acetylcholinesterase (AChE), butyrylcholinesterase (BChE), and monoamine oxidase‐B (MAO‐B), as well as antioxidant potential via on‐line HPLC‐based assays. Structures were confirmed by ^1^H‐NMR, ^13^C‐NMR (APT), and elemental analysis. Several compounds showed notable, micromolar‐range inhibitory activity, though less potent than the reference drugs. Kinetic analysis showed that the leading compounds inhibited their respective enzymes via a mixed‐type mechanism. Compound **1** showed the strongest AChE inhibition (IC_50_ = 0.777 ± 0.014 µM), compound **9b** the highest BChE inhibition (IC_50_ = 0.659 ± 0.005 µM), and compound **9c** the most potent MAO‐B inhibition (IC_50_ = 2.431 ± 0.003 µM). Compound **8a** showed the highest CUPRAC copper‐reducing capacity, while **7c** displayed the strongest DPPH radical‐scavenging activity. Liposomal formulations of **4c**, **7c**, and **8a** exhibited enhanced antioxidant responses relative to their free forms. In silico ADME predictions (SwissADME) indicated favorable drug‐likeness and blood–brain barrier permeation for the compact scaffold (compound **1**) and the piperazine‐based hybrids, whereas the larger bis‐conjugated derivatives were limited by high polarity and molecular weight. Overall, these benzimidazolone‐based hybrids represent promising multi‐target candidates for AD drug development.

## 1 Introduction

Alzheimer's disease (AD) is a multifactorial neurodegenerative disorder characterized by progressive cognitive decline, driven by cholinergic deficit, amyloid‐β aggregation, tau hyperphosphorylation, oxidative stress, and neuroinflammation (Breijyeh and Karaman 2020; Safiri et al. 2024; Tenchov et al. 2024). Cholinesterase inhibitors targeting acetylcholinesterase (AChE) and butyrylcholinesterase (BChE) remain the mainstay of symptomatic treatment but provide only modest benefit and fail to halt disease progression (Chen et al. 2022; Grabowska et al. 2025; Huang et al. 2022; Stanciu et al. 2019; Walczak‐Nowicka and Herbet 2021). Monoamine oxidase‐B (MAO‐B), upregulated in astrocytes surrounding amyloid plaques, further drives oxidative stress and neuronal damage through reactive oxygen species generation during monoamine metabolism (Gaspar et al. 2011; Giovannuzzi et al. 2024; Ribeiro et al. 2025; Rollas and Küçükgüzel 2007). Given this multifactorial pathology, simultaneous modulation of cholinesterase activity, MAO‐B‐mediated oxidative stress, and radical scavenging represents a rational therapeutic strategy (Saggu et al. 2024), motivating growing interest in multi‐target‐directed ligands (MTDLs) that integrate these activities within a single molecular framework (Beretti et al. 2025; Dash et al. 2025; Fanlo‐Ucar et al. 2024). However, most reported AD candidates remain single‐target agents with a suboptimal potency–selectivity–pharmacokinetic balance, leaving the design of effective multifunctional scaffolds an important open challenge.

Beyond cholinergic and monoaminergic dysfunction, oxidative stress is itself a central driver of AD pathology: elevated reactive oxygen species levels promote amyloid‐β aggregation, tau hyperphosphorylation, mitochondrial dysfunction, and neuronal apoptosis. Consequently, compounds capable of simultaneously inhibiting AChE, BChE, and MAO‐B while also scavenging free radicals or enhancing antioxidant capacity are of particular interest, since such multifunctional activity addresses cholinergic deficit and monoamine‐linked oxidative damage together, offering a more comprehensive therapeutic rationale than single‐target cholinesterase or antioxidant agents alone.

**Figure 1:**
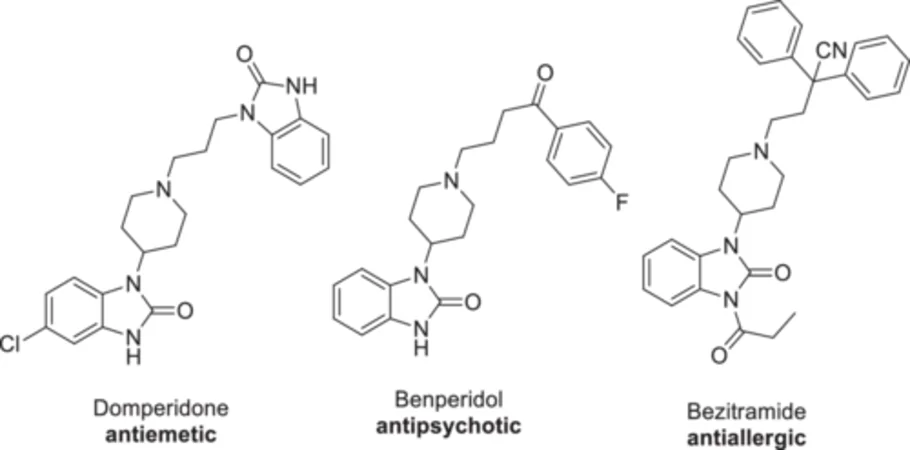
Some clinically used benzimidazolone‐containing drugs.

Benzimidazolone derivatives are widely recognized as privileged medicinal‐chemistry scaffolds due to their structural versatility and favorable interaction profiles with biological targets (Çalişkan et al. 2024, 2025; Güven et al. 2026; Güven, Menteşe, Yılmaz, et al. 2025; Menteşe et al. 2024; Yilmaz et al. 2026), reported to possess anticancer (Rode et al. 2015), anti‐HIV (Pribut et al. 2019), antiviral (Yu et al. 2006), antidiabetic (Abbas et al. 2015), and antimicrobial (Patel et al. 2014) activity, and are present in clinically used drugs including domperidone (Brogden et al. 1982), benperidol (Schwarz et al. 2005), and benzitramide (Rode et al. 2015) (Figure 1).

**Figure 2:**
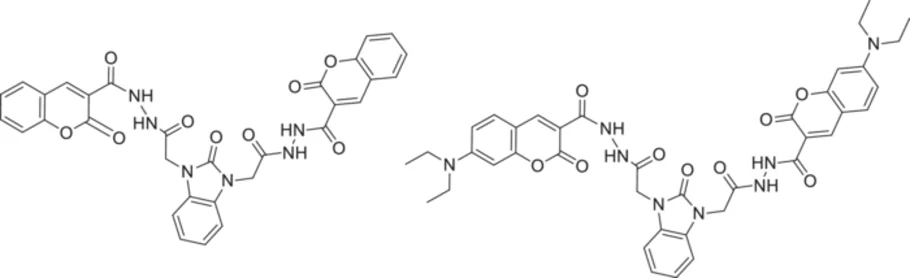
Two benzimidazolone based AChE inhibitors were shown.

These derivatives have also shown promising cholinesterase‐inhibitory activity relevant to AD (Figure 2), yet remain relatively underexplored in AD‐focused biochemical studies, representing a gap in the current literature (Güven, Menteşe, Bilgin Sökmen, et al. 2025; Intagliata et al. 2019; Menteşe et al. 2025).

**Figure 3:**
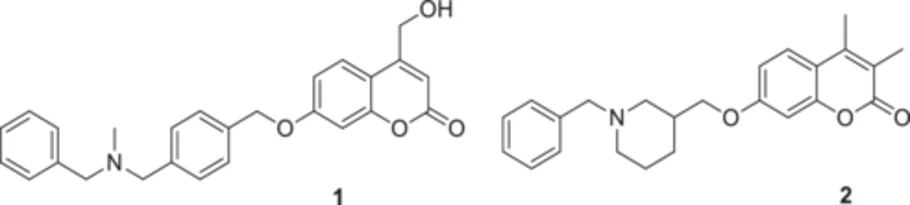
Coumarin‐based AChE‐MAO B inhibitors.

Coumarin derivatives are similarly well documented as potent cholinesterase inhibitors and, in several cases, as dual AChE/MAO‐B inhibitors that engage both the catalytic site and the FAD‐binding domain (Figure 3) (Abu‐Aisheh et al. 2019; Ekström et al. 2022; Gouvea et al. 2026; S. Singh et al. 2025).

Triazole‐containing pharmacophores, found in drugs such as anastrozole, fluconazole, and voriconazole, likewise contribute metabolic stability and cholinesterase‐inhibitory activity when incorporated into hybrid structures (Bekircan et al. 2022; Elbadawi et al. 2025; Khan et al. 2023; Li et al. 2023; A. Singh et al. 2023; Sobha et al. 2025; Yazdani et al. 2019).

Molecular hybridization, which combines distinct pharmacophores within a single framework to achieve synergistic multi‐target activity, is a well‐established MTDL design strategy (Gontijo et al. 2020; Hossain and Hussain 2025; Zhou et al. 2019). However, benzimidazolone‐based hybrids that concurrently incorporate coumarin and triazole moieties to target both cholinesterases and MAO‐B remain largely unexplored, representing a clear gap in the current literature.

In the present study, we report the design, synthesis, and biological evaluation of a new series of benzimidazolone‐based hybrids incorporating coumarin, triazole, and piperazine moieties as multi‐target agents for AD. The synthesized compounds were evaluated for inhibitory activity against AChE, BChE, and MAO‐B, together with their antioxidant capacity, and structure–activity relationships (SAR) were analyzed to guide rational design. Molecular docking against the corresponding target enzymes and in silico ADME/drug‐likeness prediction (SwissADME) were additionally performed to relate the observed multi‐target activity to plausible ligand–enzyme interactions and CNS developability.

## 2 Results and Discussion

### 2.1 Chemistry

**Scheme 1:**
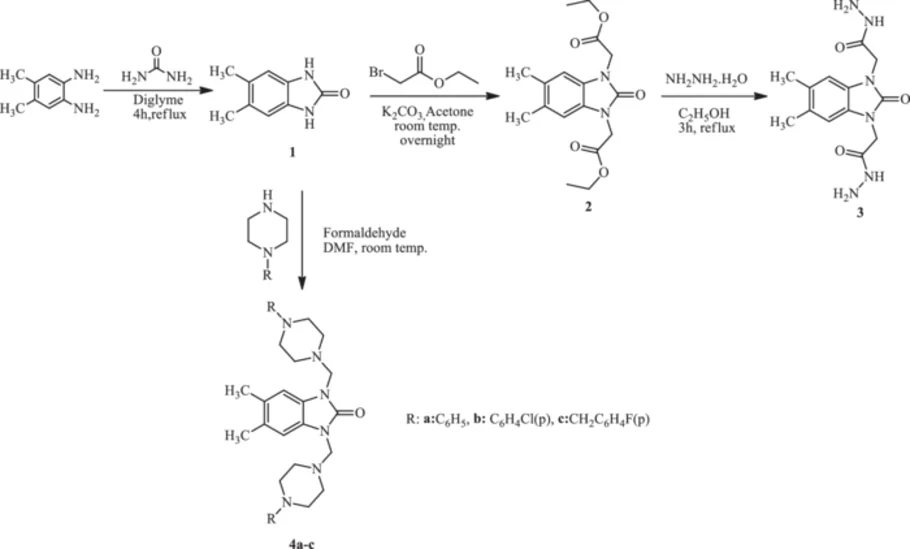
Synthesis of compounds **1, 2, 3,** and **4a–c**.

**Scheme 2:**
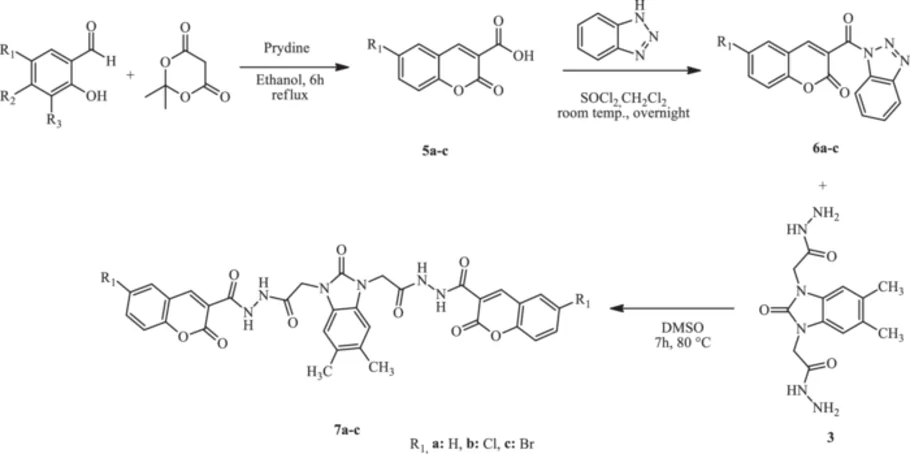
Synthesis of compounds **5–7a–c**.

**Scheme 3:**
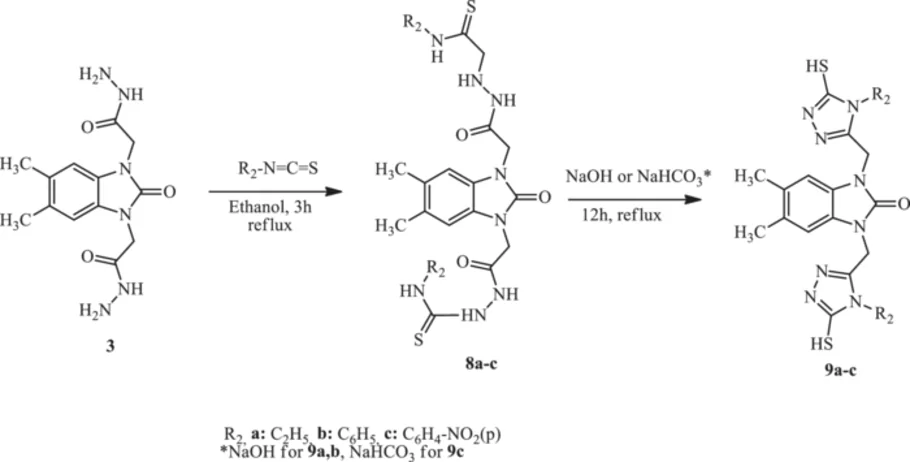
Synthesis of compounds **8–9a–c**.

The synthesis of the target benzimidazolone‐based hybrid compounds was accomplished through a multistep synthetic pathway, as outlined in Schemes 1, 2, 3. The synthetic route was initiated with the preparation of 5,6‐dimethyl‐1*H*‐benzo[d]imidazol‐2(3*H*)‐one (**1**) via the condensation of 4,5‐dimethyl‐o‐phenylenediamine with urea under reflux conditions. Subsequent alkylation of compound **1** with ethyl bromoacetate in the presence of potassium carbonate afforded the diester derivative (**2**), which was further converted into the corresponding dihydrazide (**3**) through reaction with hydrazine hydrate. Piperazine‐containing derivatives (**4a–c**) were synthesized via a Mannich‐type reaction involving compound **1**, formaldehyde, and the corresponding substituted piperazines (Scheme 1) (Güven et al. 2023; Güven, Menteşe, Bilgin Sökmen, et al. 2025; Menteşe et al. 2021).

In parallel, coumarin‐based intermediates (**5a–c**) were prepared from salicylaldehyde derivatives and Meldrum's acid, followed by activation with benzotriazole to yield intermediates (**6a–c**). The literature demonstrates that benzotriazole is an easily leaving group with the capacity to facilitate a variety of chemical transformations. To obtain coumarin‐containing benzotriazole derivatives (**6a–c**), we have reacted compound **5a–c** with 1H‐benzotriazole in dichloromethane in the presence of SOCl_2_. These intermediates were subsequently coupled with compound **3** to afford the benzimidazolone–coumarin hybrid derivatives (**7a–c**) (Çalişkan et al. 2024; Menteşe et al. 2016) (Scheme 2).

Further functionalization of compound **3** with isothiocyanates yielded intermediates (**8a–c**), which were cyclized under basic conditions to produce the corresponding triazole derivatives (**9a–c**) (Scheme 3).

The spectral data of the newly synthesized compounds are consistent with the proposed structures. In the ^1^H‐NMR spectra of compound **1**, the signals corresponding to the CH_3_ protons on the benzimidazolone 5th and 6th position were observed at 2.14 ppm, and the NH signals were observed at 10.33 ppm. Aromatic CH protons (4th and 7th positions) were seen at 6.88 ppm. When the ^13^C‐APT spectra of compound **1** were investigated, C═O group signals in the benzimidazolone ring were seen at 155.87 ppm. It was observed that the signals belonging to the CH_3_ on benzimidazolone 5th and 6th position were seen at about 19.85 ppm. Other aromatic CH and quaternary carbon atoms were observed at 120.21 and 110.00 ppm, respectively. In the ^1^H‐NMR spectra of compound **2**, the NCH_2_ signal was shown at 4.65 ppm and the CH_2_ and CH_3_ signals coming from ethoxy were shown at 4.13 and 1.88 ppm, respectively. No NH signal (coming from compound **1**) was seen at about 10.10 ppm, this confirms the occurrence of the alkylation reaction. In ^13^C‐NMR spectra of this compound, C═O signal coming from alkylation and NCH_2_ were shown at 168.52 and 42.38 ppm, respectively. OCH2 and CH3 signals coming from the ethoxy group were shown at 61.51 and 14.47 ppm, respectively. In the ^1^H‐NMR spectra of compound **3**, NH and NH_2_ signals at 9.25 and 4.29 ppm, respectively, were formed. Also, OCH_2_CH_3_ signals disappeared in ^1^H‐NMR and ^13^C‐NMR spectra of this compound. In ^1^H‐NMR spectra of compound **4a–c**, two piperazine CH_2_ signals were shown at 2.50–2.70 and 3.00–3.37 ppm. The NCH_2_ signal of this compound was shown at about 4.60 ppm. In ^13^C‐NMR spectra of these compounds, piperazine CH_2_ carbon atoms were seen at about 48–50 and 50–53 ppm and NCH_2_ carbon were shown at about 62.50 ppm. Other aromatic and aliphatic carbons were observed at a suitable range in ^13^C‐NMR spectra of these compounds. In 1H‐NMR spectra of compound **7a–c,** the NH2 signal (coming from compound **3**) disappeared, and two new NH signals at about 11.30–11.10 and 10.64–10.60 ppm and a new coumarin C3H signal at about 8.80 ppm were formed. In ^13^C‐NMR spectra of these compounds, new C═O signals coming from the coumarin cycle were shown at about 159 and 160 ppm. Coumarin C_3_ and C_4_H carbons were seen at about 153–154 and 147–149 ppm, respectively. In ^1^H‐NMR spectra of isothiocyanate derivatives (compound **8a–c),** three NH signals were shown between 8.00 and 10.50 ppm and NCH_2_ signal was seen at about 4.50 ppm. In ^13^C‐NMR spectra of these compounds, C═S signals were shown at about 180–187 ppm (not detected for compound **8b)** and NCH_2_ was shown at about 42 ppm. In ^1^H‐NMR spectra of triazole derivatives (compounds **9a–c**), NH signals (coming from isothiocyanate) disappeared, and a new SH signal was formed at about 14 ppm. In ^13^C‐NMR spectra of these compounds, two triazol C═N carbon atoms were shown at about 148 (triazol‐C_3_) and 168 (triazol C_5_) ppm. In addition, all compounds showed suitable elemental analyses results with calculated data.

### 2.2 Enzyme Inhibition Results

The synthesized compounds were evaluated for their inhibitory activities against AChE, BChE, and MAO‐B enzymes, and the results are summarized in Table 1.

**Table 1:** The IC_50_ values of AChE, BChE, and MAO‐B enzymes test results of all the newly synthesized compounds.

	AChE	BChE	MAO‐B
Compounds	IC_50_ (µM) ± SD	IC_50_ (µM) ± SD	IC_50_ (µM) ± SD
**1**	0.777 ± 0.014	12.702 ± 0.006	4.213 ± 0.014
**2**	7.019 ± 0.014	18.709 ± 0.003	7.040 ± 0.005
**3**	9.419 ± 0.011	23.766 ± 0.014	8.239 ± 0.008
**4a**	7.497 ± 0.006	7.037 ± 0.003	6.313 ± 0.003
**4b**	23.697 ± 0.014	0.946 ± 0.013	9.387 ± 0.003
**4c**	10.990 ± 0.004	8.750 ± 0.006	3.407 ± 0.005
**7a**	1.324 ± 0.013	8.594 ± 0.005	22.012 ± 0.014
**7b**	7.932 ± 0.002	20.605 ± 0.006	6.325 ± 0.002
**7c**	3.710 ± 0.003	3.340 ± 0.007	3.210 ± 0.013
**8a**	21.936 ± 0.001	21.520 ± 0.006	8.558 ± 0.010
**8b**	23.128 ± 0.002	22.049 ± 0.013	21.008 ± 0.003
**8c**	19.600 ± 0.009	7.259 ± 0.007	6.205 ± 0.005
**9a**	11.746 ± 0.003	3.263 ± 0.004	3.153 ± 0.014
**9b**	16.720 ± 0.008	0.659 ± 0.005	6.104 ± 0.015
**9c**	8.915 ± 0.012	8.510 ± 0.007	2.431 ± 0.003
**Donepezil**	0.007 ± 0.001	0.010 ± 0.001	—
**Selegiline**	—	—	0.009 ± 0.002

*Note:* Donepezil and Selegiline were used as standard inhibitors. The results of all studied compounds are expressed as the mean ± SD in triplicate. IC_50_ value, µM.

#### 2.2.1 In Vitro AChE Inhibition Studies and Kinetic Results

**Figure 4:**
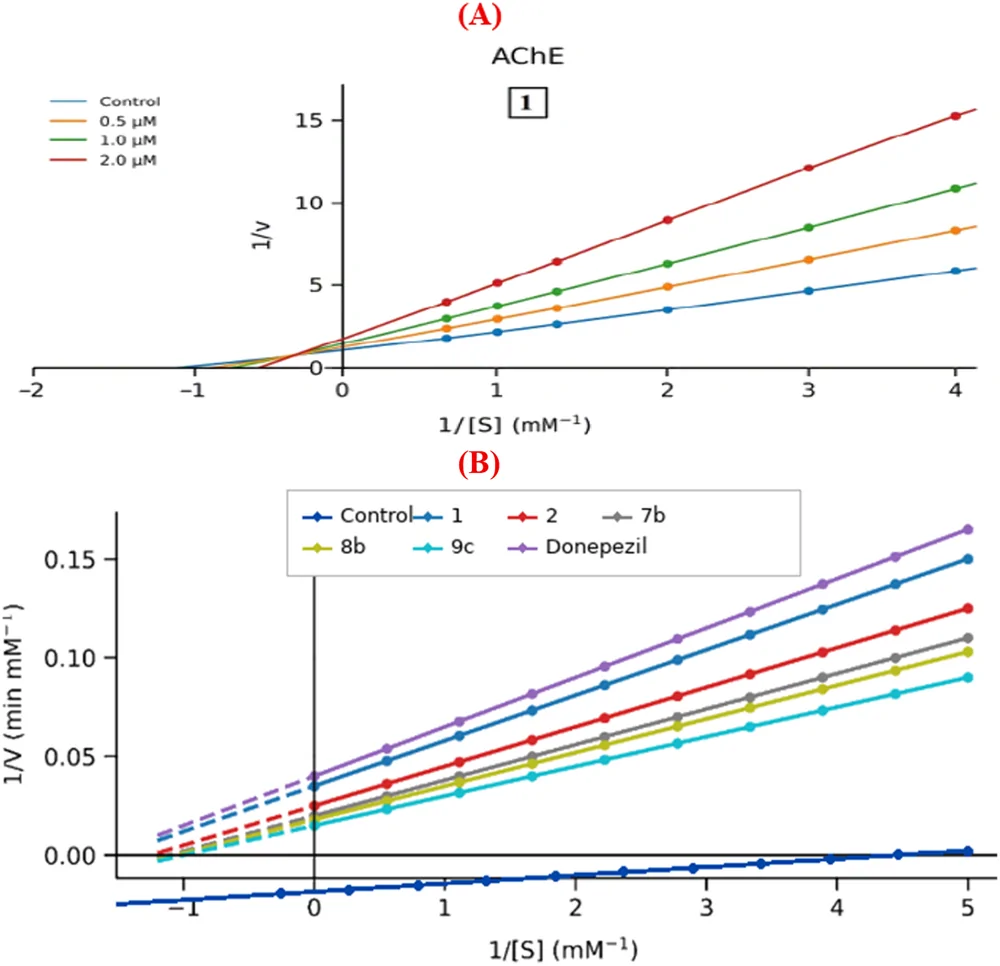
Lineweaver–Burk plots for AChE inhibition. (A) Representative individual plot for compound 1 (0, 0.5, 1.0, and 2.0 µM), showing the characteristic mixed‐type pattern: increasing slope and y‐intercept with a common intersection point in the second quadrant. (B) Comparative plots for **1, 2, 7b, 8b, 9c,** and Donepezil. Kinetic parameters derived from these plots, including the control *K*_m_/*V*_max_ values and the inhibition constants (*K*_i_, α*K*_i_) confirming the mixed‐type mechanism are reported in Table 2.

The newly synthesized compounds were evaluated for their AChE inhibitory potential. Percentage inhibition values were plotted against inhibitor concentrations separately for each compound. Among the synthesized molecules, compound **1** exhibited the most potent AChE inhibitory activity, with an IC_50_ value of 0.777 ± 0.014 µM (Table 1), whereas the reference drug donepezil showed an IC_50_ value of 0.007 ± 0.001 µM. Based on their AChE inhibitory activities, the tested compounds were ranked from most to least active as follows: **Donepezil** > **1** > **7a** > **7c** > **2** > **7b** > **4a** > **9c** > **3** > **4c** > **9a** > **9b** > **8c** > **8a** > **8b** > **4b** (Table 1). To determine the mode of enzyme inhibition, AChE activity was further analyzed using Lineweaver–Burk plots, employing kinetic data obtained at different concentrations of acetylthiocholine iodide (ATC) in the absence and presence of each inhibitor (Table 2, Figure 4) (Lineweaver and Burk 1934). Lineweaver–Burk analysis, together with the *K*_i_ and α*K*_i_ values derived from global nonlinear fitting, revealed that all synthesized compounds and donepezil inhibited AChE via a mixed‐type mechanism, engaging both the free enzyme and the enzyme–substrate complex (Table 2). Among the tested compounds, compound **1** exhibited the strongest inhibitory affinity for the enzyme, as reflected by its low *K*_i_ value (0.55 µM), consistent with its potent IC_50_. In contrast, compounds such as **4b** and **8b**, which displayed markedly higher *K*_i_ values (18.50 and 19.50 µM, respectively), showed correspondingly weaker inhibitory potency (Table 2).

**Table 2:** Type of AChE inhibition of the synthesized compounds and Donepezil with their *K*_m_ and *V*_max_ values.

Compound	Inhibitor conc. (µM)	Type of inhibition	Control *K*_m_ (mM)	Control *V*_max_ (mM/min)	*K*_m_ (mM)	*V*_max_ (mM/min)	*K*_i_ (µM)	α*K*_i_	IC_50_ (µM)	*R* ^2^
**1**	1.0	Mixed‐type inhibition	0.95	245	4.056	203.436	0.55	3.20	0.777	0.998
**2**	10	Mixed‐type inhibition	0.95	245	1.368	179.161	4.80	1.80	7.019	0.998
**3**	10	Mixed‐type inhibition	0.95	245	1.654	184.439	6.20	1.90	9.419	0.998
**4a**	10	Mixed‐type inhibition	0.95	245	1.389	191.644	5.10	1.70	7.497	0.998
**4b**	10	Mixed‐type inhibition	0.95	245	0.340	69.227	18.50	0.55	23.697	0.991
**4c**	10	Mixed‐type inhibition	0.95	245	0.356	77.463	8.10	0.65	10.990	0.998
**7a**	10	Mixed‐type inhibition	0.95	245	2.835	164.924	0.95	2.50	1.324	0.998
**7b**	10	Mixed‐type inhibition	0.95	245	1.737	181.252	5.80	2.00	7.932	0.998
**7c**	10	Mixed‐type inhibition	0.95	245	1.993	177.000	2.60	2.10	3.710	0.998
**8a**	10	Mixed‐type inhibition	0.95	245	0.563	124.012	17.00	0.90	21.936	0.998
**8b**	10	Mixed‐type inhibition	0.95	245	0.404	81.388	19.50	0.70	23.128	0.997
**8c**	10	Mixed‐type inhibition	0.95	245	0.538	110.990	15.50	0.85	19.600	0.998
**9a**	10	Mixed‐type inhibition	0.95	245	0.355	74.484	9.50	0.62	11.746	0.998
**9b**	10	Mixed‐type inhibition	0.95	245	0.436	85.459	13.20	0.75	16.720	0.998
**9c**	10	Mixed‐type inhibition	0.95	245	0.317	65.168	6.90	0.50	8.915	0.988
**Donepezil**	0.01	Mixed‐type (reference inhibitor)	0.95	245	8.873	180.380	0.004	4.50	0.007	0.998

#### 2.2.2 In Vitro BChE Inhibition Studies and Kinetic Results

**Figure 5:**
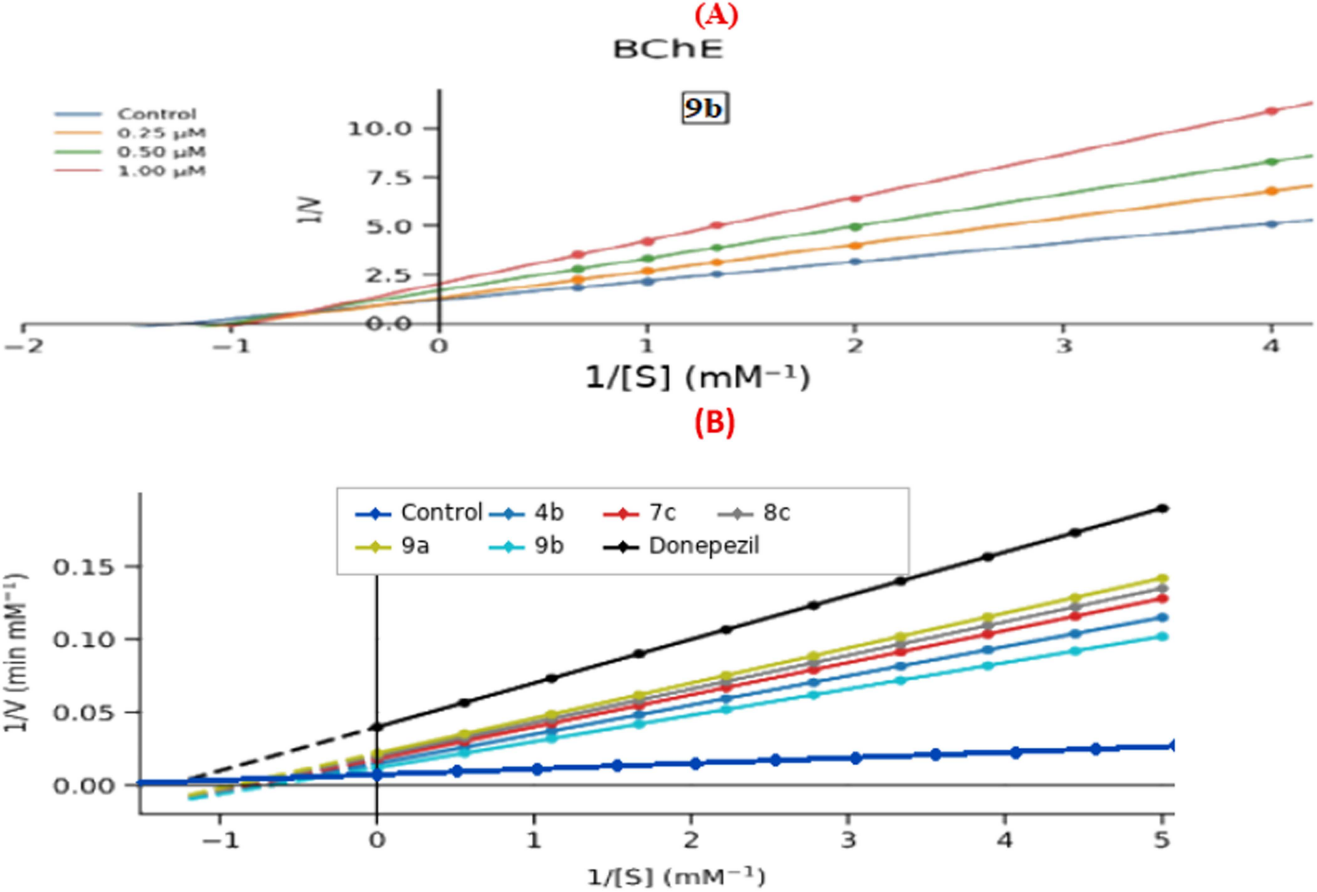
Lineweaver–Burk plots for BChE inhibition. (A) Representative individual plot for compound 9b (0, 0.25, 0.50, and 1.00 µM), showing the characteristic mixed‐type pattern. (B) Comparative plots in the presence of **4b, 7c, 8c, 9a, 9b,** compounds and Donepezil. The plots were generated using at least three inhibitor concentrations, and kinetic parameters were extracted accordingly. Kinetic parameters derived from these plots, including the control *K*_m_/*V*_max_ values and the inhibition constants (*K*_i_, α*K*_i_) confirming the mixed‐type mechanism are reported in Table 3.

All newly synthesized compounds were evaluated for their inhibition potential against BChE. The percentage relative activities versus inhibitor concentrations were plotted separately for each compound. Among the tested compounds, compound **9b** exhibited the most potent inhibitory activity, with IC_50_ value 0.659 ± 0.005 µM (Table 1). The BChE inhibitory effects of the compounds are listed from the most active to the least active as follows: **Donepezil** > **9b** > **4b** > **7c** > **9a** > **8c** > **4a** > **7a** > **9c** > **4c** > **1** > **2** > **7b** > **8a** > **8b** > **3** (Table 1). Determination of the type of enzyme inhibition of synthesized compounds and Donepezil, their BChE activity was analyzed by Lineweaver–Burk plots using data derived from enzyme assay containing different concentrations of BTC in the absence or presence of each inhibitor (Table 3, Figure 5). The Lineweaver–Burk analysis, supported by the *K*_i_ and α*K*_i_ values obtained from global fitting, indicated that all compounds and donepezil inhibited BChE via a mixed‐type mechanism (Table 3, Figure 5). Among the tested compounds, **9b** exhibited the strongest inhibitory affinity, reflected by its low *K*_i_ value (0.45 µM), consistent with its potent IC50 (0.659 µM); in contrast, compounds **8b** and **3**, with the highest *K*_i_ values (18.5 and 18.7 µM, respectively), displayed correspondingly weaker inhibitory potency (Table 3, Figure 5).

**Table 3:** Type of BChE inhibition of the synthesized compounds and Donepezil with their *K*_m_ and *V*_max_ values.

Compound	Inhibitor conc. (µM)	Type of inhibition	Control *K*_m_ (mM)	Control *V*_max_ (mM/min)	*K*_m_ (mM)	*V*_max_ (mM/min)	*K*_i_ (µM)	α*K*_i_	IC_50_ (µM)	*R* ^2^
**1**	10	Mixed‐type inhibition	0.85	220	0.328	70.748	10.8	0.55	12.702	0.998
**2**	10	Mixed‐type inhibition	0.85	220	0.345	75.781	15.5	0.60	18.709	0.996
**3**	10	Mixed‐type inhibition	0.85	220	0.541	109.444	18.7	0.80	23.766	0.996
**4a**	10	Mixed‐type inhibition	0.85	220	1.973	191.353	4.8	2.10	7.037	0.998
**4b**	1.0	Mixed‐type inhibition	0.85	220	3.128	190.201	0.65	3.40	0.946	0.998
**4c**	10	Mixed‐type inhibition	0.85	220	0.421	84.098	6.7	0.70	8.750	0.998
**7a**	10	Mixed‐type inhibition	0.85	220	1.943	167.696	5.5	2.00	8.594	0.998
**7b**	10	Mixed‐type inhibition	0.85	220	0.479	94.836	13.8	0.75	20.605	0.998
**7c**	5.0	Mixed‐type inhibition	0.85	220	2.037	190.988	2.2	2.30	3.340	0.998
**8a**	10	Mixed‐type inhibition	0.85	220	0.405	80.618	17.0	0.65	21.520	0.995
**8b**	10	Mixed‐type inhibition	0.85	220	0.549	107.512	18.5	0.80	22.049	0.997
**8c**	10	Mixed‐type inhibition	0.85	220	2.025	200.800	5.2	2.40	7.259	0.998
**9a**	5.0	Mixed‐type inhibition	0.85	220	1.808	187.946	2.0	2.20	3.263	0.998
**9b**	1.0	Mixed‐type inhibition	0.85	220	3.314	157.392	0.45	3.60	0.659	0.998
**9c**	10	Mixed‐type inhibition	0.85	220	0.416	82.021	6.5	0.70	8.510	0.998
**Donepezil**	0.01	Mixed‐type (reference inhibitor)	0.85	220	6.857	177.663	0.005	4.80	0.010	0.998

#### 2.2.3 In Vitro MAO‐B Inhibition Studies and Kinetic Results

**Figure 6:**
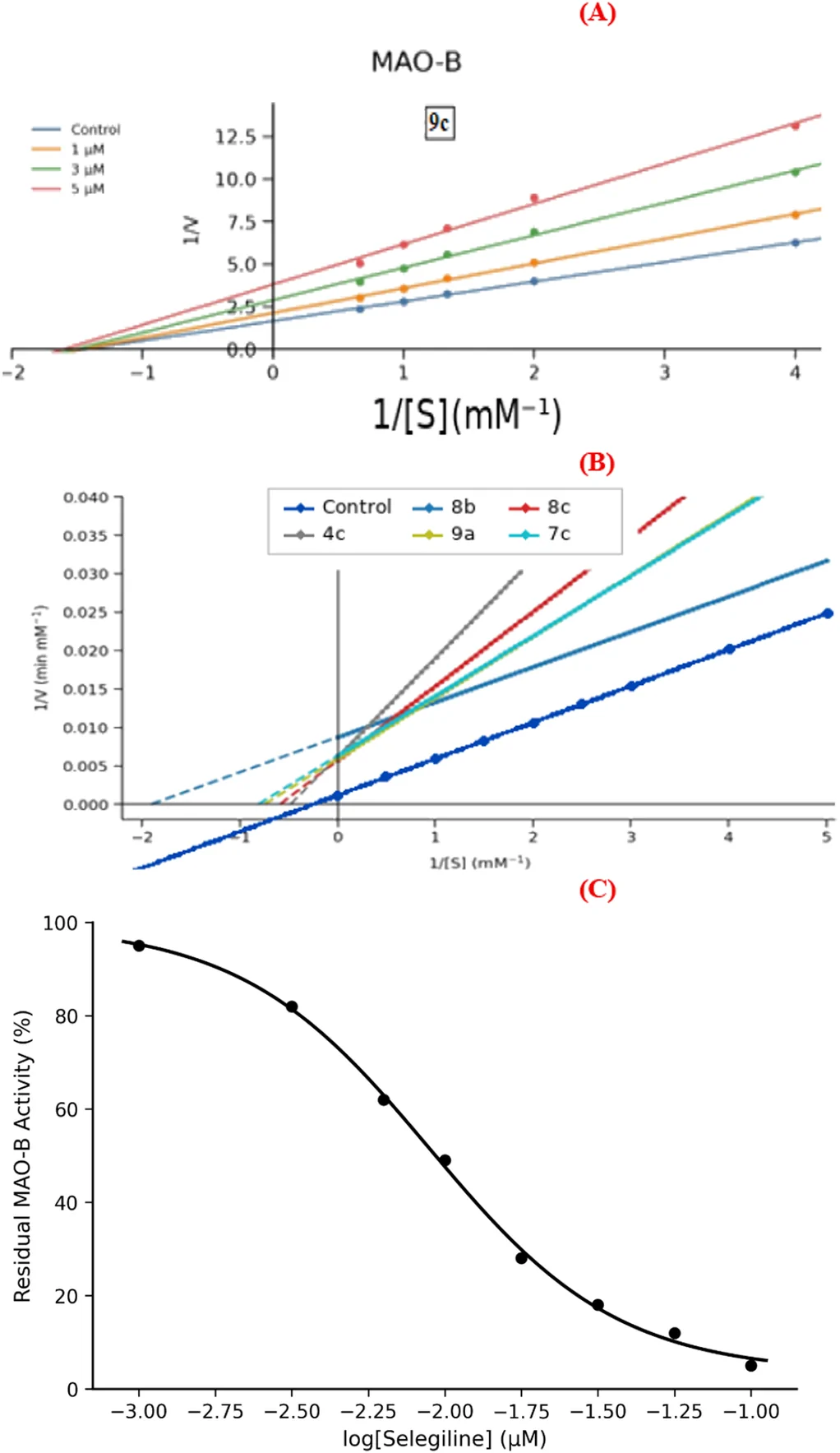
Lineweaver–Burk plots for MAO‐B inhibition. (A) Representative individual plot for compound **9c** (0, 1, 3, and 5 µM), showing the characteristic mixed‐type pattern. (B) Comparative plots for **8b, 8c, 4c, 9a, 7c,** and Selegiline. Kinetic parameters derived from these plots, including the control *K*_m_/*V*_max_ values and the inhibition constants (*K*_i_, α*K*_i_), confirming the irreversible reference mechanism of selegiline versus the mixed‐type mechanism of the tested compounds, are reported in Table 4. (C) Dose−response curve for the reference inhibitor selegiline, showing residual MAO‐B activity (%) as a function of log[selegiline], used to determine its IC50 by nonlinear regression (log[inhibitor] vs. response, variable slope).

The synthesized compounds were assessed for their inhibitory activities against MAO‐B at concentrations of 10^−3 ^M. The percentage inhibitions of these compounds on both enzymes, along with reference drug selegiline, are presented in Table 1. Notably, the synthesized compounds demonstrated inhibition of the MAO‐B enzyme over 50% at the 10^−3^ concentration. However, the synthesized compounds did not exhibit more than 50% inhibition of MAO‐A. This observation suggests a higher affinity of the compounds for MAO‐B, indicating a qualitative preference for inhibiting MAO‐B over MAO‐A. In contrast to the MAO‐B results, none of the synthesized compounds displayed more than 50% inhibition toward MAO‐A at 10^−3^ M concentration in the preliminary screening. This indicates that the newly synthesized benzimidazolone‐based hybrids exhibit weak affinity toward MAO‐A and thus demonstrate favorable selectivity for MAO‐B, consistent with their structural features and redox‐active heterocycles. Therefore, detailed kinetic characterization was not pursued for MAO‐A. The MAO‐B inhibitory effects of the compounds are listed from the most active to the least active molecules as follows: **Selegiline** > **9c** > **9a** > **7c** > **4c** > **1** > **9b** > **8c** > **4a** > **7b** > **2** > **3** > **8a** > **4b** > **8b** > **7a** (Table 1). To determine the mode of enzyme inhibition, MAO‐B activity was further analyzed using Lineweaver–Burk plots, employing kinetic data obtained at different concentrations of MAO‐B in the absence and presence of each inhibitor (Table 4, Figure 6). Lineweaver–Burk analysis, together with the *K*_i_ and α*K*_i_ values obtained from global fitting, revealed that all synthesized compounds and selegiline inhibited MAO‐B via a mixed‐type mechanism (Table 4), in contrast to the irreversible mechanism characteristic of selegiline itself. Among the tested compounds, **9c** exhibited the strongest inhibitory affinity, reflected by its low *K*_i_ value (1.4 µM), consistent with its potent IC_50_ (2.431 µM); in contrast, compound **8b**, with the highest *K*_i_ value (17.5 µM), displayed correspondingly weaker inhibitory potency (Table 4).

**Table 4:** Type of MAO‐B inhibition of the synthesized compounds and Selegiline with their *K*_m_ and *V*_max_ values.

Compound	Inhibitor conc. (µM)	Type of inhibition	Control *K*_m_ (mM)	Control *V*_max_ (mM/min)	*K*_m_ (mM)	*V*_max_ (mM/min)	*K*_i_ (µM)	α*K*_i_	IC_50_ (µM)	*R* ^2^
**1**	5.0	Mixed‐type inhibition	0.90	210	1.460	148.214	2.5	1.80	4.213	0.998
**2**	10	Mixed‐type inhibition	0.90	210	1.946	169.601	5.0	2.20	7.040	0.998
**3**	10	Mixed‐type inhibition	0.90	210	1.725	170.053	5.8	2.00	8.239	0.998
**4a**	10	Mixed‐type inhibition	0.90	210	1.463	157.763	4.5	1.90	6.313	0.998
**4b**	10	Mixed‐type inhibition	0.90	210	1.265	169.817	7.5	1.60	9.387	0.998
**4c**	5.0	Mixed‐type inhibition	0.90	210	2.080	161.824	1.8	2.80	3.407	0.998
**7a**	20	Mixed‐type inhibition	0.90	210	0.432	87.094	18.0	0.70	22.012	0.998
**7b**	10	Mixed‐type inhibition	0.90	210	0.975	141.887	5.2	1.20	6.325	0.998
**7c**	5.0	Mixed‐type inhibition	0.90	210	1.233	158.008	2.0	1.60	3.210	0.998
**8a**	10	Mixed‐type inhibition	0.90	210	1.266	160.457	6.8	1.40	8.558	0.998
**8b**	20	Mixed‐type inhibition	0.90	210	0.524	114.309	17.5	0.85	21.008	0.996
**8c**	10	Mixed‐type inhibition	0.90	210	1.701	175.468	4.8	2.00	6.205	0.998
**9a**	5.0	Mixed‐type inhibition	0.90	210	1.339	168.288	1.9	1.70	3.153	0.998
**9b**	10	Mixed‐type inhibition	0.90	210	1.388	157.518	5.0	1.80	6.104	0.998
**9c**	3.0	Mixed‐type inhibition	0.90	210	1.546	153.038	1.4	2.10	2.431	0.998
**Selegiline**	0.01	Irreversible (reference inhibitor)	0.90	210	9.814	167.205	0.003	—	0.009	0.998

### 2.3 Lipozomal Forms and On‐Line HPLC Analysis

**Figure 7:**
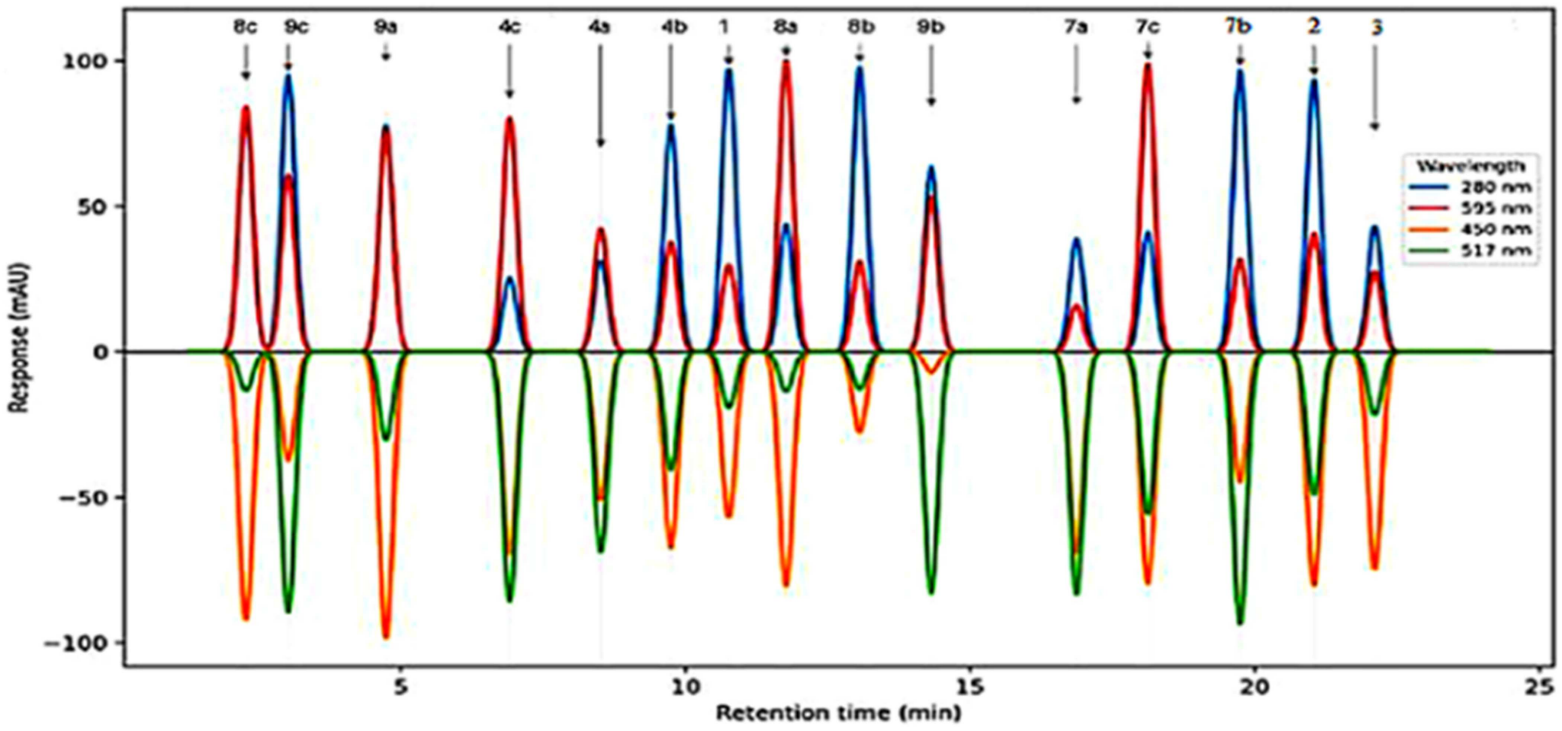
Peak chromatogram overlap of 15 synthesized compounds at four wavelengths (280, 595, 517, 450 nm).

**Figure 8:**
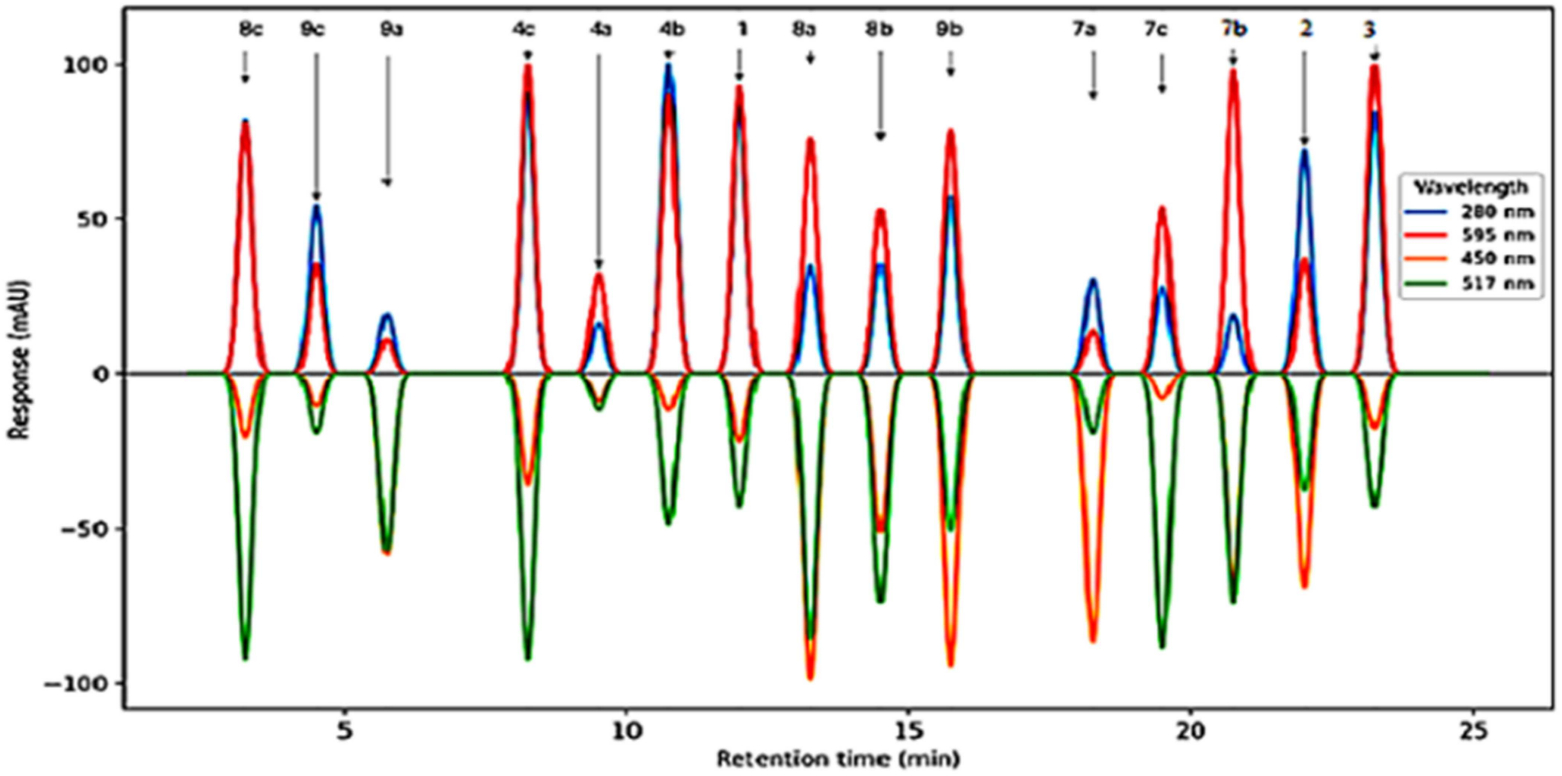
Peak chromatogram overlap of liposomal forms of synthesized compounds at four wavelengths (280, 595, 517, 450 nm).

For all of the synthesized compounds, liposomes were prepared identically by the thin‑film hydration–extrusion workflow: phosphatidylcholine and cholesterol (≈7:3, mol:mol) were dissolved in chloroform:methanoland evaporated under reduced pressure to form a uniform lipid film, the film was vacuum‑dried and hydrated above the lipid Tm with an aqueous medium containing each compound to generate multilamellar dispersions, which were then nano‑sized by serial extrusion through polycarbonate membranes (400 → 200 → 100 nm) to yield near‑monodisperse nanoliposomes (~100–150 nm) according to nanoliposome protocol described in the methodological literature (Zhang 2017) The synthesized compounds were prepared at 1.000 mg/mL in DMSO and their liposomal forms in Aqua screened for antioxidant activity using online HPLC–FRAP, HPLC–CUPRAC, and HPLC–DPPH methodologies coupled post‑column to a RP‐18 separation. UV–DAD detection was performed at 280 nm for chromatographic peaks and at 595, 517, and 450 nm for FRAP, DPPH, and CUPRAC responses, respectively (Apak et al. 2004; Benzie and Strain 1999). Validation included LOD and LOQ in accordance with ICH principles (Armbruster and Pry 2008). Synthetic concentrations and detection‑limit values are reported per compound with three‑decimal precision. The antioxidant profiles of the synthesized benzimidazolone‐based hybrid compounds were evaluated using online HPLC–FRAP, HPLC–DPPH, and HPLC–CUPRAC post‐column systems. In this approach, each compound was first separated on an RP‐HPLC system and subsequently reacted in real time with the corresponding antioxidant reagents through a post‐column reaction coil, enabling simultaneous signal acquisition at 280, 595, 517, and 450 nm. The overlaid chromatograms of both the pure compounds and their liposomal formulations are presented in Figures 7 and 8, demonstrating their chromatographic separation as well as their FRAP, DPPH, and CUPRAC responses. Retention times, concentration data, detection limits, and validation parameters provided in Supporting Information S1: Tables 1–6. confirm the reliability and analytical robustness of the method. Comparative evaluation of antioxidant activities across all three post‐column systems revealed that compound **7c** exhibited the highest overall activity, followed by **8a** and **4c**, which also produced strong responses in multiple assays. Moreover, the liposomal forms of selected compounds (notably **4c**, **7c**, and **8a**) showed enhanced signal intensities, indicating improved antioxidant accessibility or stability. Collectively, these findings demonstrate that the synthesized hybrids possess significant redox‐active potential and highlight the effectiveness of the online HPLC platform for real‐time discrimination of their antioxidant behaviors.

### 2.4 SAR Analysis

The inhibitory activities of the synthesized benzimidazolone‐based hybrids against AChE, BChE, and MAO‐B revealed clear structure–activity trends, highlighting the impact of molecular hybridization on multi‐target potency.

#### 2.4.1 AChE Inhibition

Among all tested compounds, compound **1** exhibited the most potent AChE inhibitory activity (IC_50_ = 0.777 µM), although it remained less potent than the reference drug donepezil. This remarkable activity can be attributed to the relatively compact benzimidazolone core, which likely facilitates efficient accommodation within the catalytic active site (CAS) of AChE. In contrast, the introduction of bulky substituents, particularly in piperazine‐containing derivatives **(4a–c)**, resulted in a noticeable decrease in AChE inhibitory activity. This reduction can be rationalized by steric hindrance, which may limit deep penetration into the narrow active‐site gorge of AChE. However, moderate activity was observed in these derivatives. The coumarin‐based hybrids **(7a–c)** displayed improved activity compared to several intermediate compounds, with **7a** and **7c** emerging as notable AChE inhibitors. Conversely, triazole derivatives **(9a–c)** demonstrated moderate AChE inhibition. Although the triazole ring provides hydrogen‐bonding capability and metabolic stability, its incorporation appears to increase molecular rigidity and reduce conformational adaptability, potentially limiting optimal positioning within the enzyme active site.

A closer look at the potency order (**1** > **7a** > **7c** > **2** > **7b** > **4a**) shows that compact, less hindered scaffolds are favored at AChE, whereas the bulky thiosemicarbazide derivatives **8a–c** were the weakest inhibitors (IC_50_ = 19.6–23.1 µM). Strikingly, the 4‐chlorophenyl piperazine **4b** was the poorest AChE inhibitor (IC_50_ = 23.697 µM) yet the strongest against BChE, revealing an inverse AChE/BChE substituent preference.

#### 2.4.2 BChE Inhibition

A distinct SAR pattern was observed for BChE inhibition. Unlike AChE, BChE possesses a larger and more flexible active site, allowing accommodation of bulkier ligands. This is reflected in the superior activity of compound **9b** (IC_50_ = 0.659 µM), which contains a triazole moiety and extended substituents. The enhanced activity of **9b** suggests that the triazole ring, in combination with hydrophobic substituents, may facilitate favorable interactions within the broader BChE gorge. Similarly, compound **4b**, a piperazine derivative, also exhibited strong BChE inhibition, indicating that flexible aliphatic linkers and basic nitrogen atoms may enhance binding through electrostatic interactions and improved conformational adaptability. In contrast, simpler benzimidazolone derivatives (e.g., compounds **1–3**) showed relatively weak BChE inhibition, further supporting the notion that increased molecular size and flexibility are advantageous for targeting BChE.

The BChE order (**9b** > **4b** > **7c** > **9a**) confirms this preference for larger ligands: the 4‐chlorophenyl derivative **4b** (IC_50_ = 0.946 µM) far exceeded its phenyl (**4a**, 7.037 µM) and 4‐fluorobenzyl (**4c**, 8.750 µM) analogues, and the triazole compounds **9a**/**9b** clearly outperformed the simple cores **1–3** (12.7–23.8 µM). Because **4b** and **9b** are weak at AChE, they behave as selective BChE inhibitors, a profile of particular relevance to advanced‐stage disease, in which BChE activity progressively predominates.

#### 2.4.3 MAO‐B Inhibition

The MAO‐B inhibitory activity demonstrated a different SAR profile, with compound **9c** (IC_50_ = 2.431 µM) emerging as the most potent inhibitor. The superior activity of this compound can be attributed to the presence of the triazole ring. The aromatic and heterocyclic nature of the triazole moiety likely enhances binding affinity within the hydrophobic substrate cavity of MAO‐B. Additionally, the presence of electron‐donating or halogen substituents may further stabilize ligand–enzyme interactions through dipole and van der Waals forces. Interestingly, compound **4c** also displayed notable MAO‐B inhibition, suggesting that piperazine‐containing derivatives may adopt conformations suitable for interaction with the enzyme cavity. The observed selectivity toward MAO‐B over MAO‐A is particularly significant and may be attributed to structural compatibility with the elongated and hydrophobic MAO‐B active site.

The MAO‐B order (**9c** > **9a** > **7c** > **4c** > **1**) indicates that triazole‐ and halogen‐bearing derivatives are consistently favored, with **4c**, **7c,** and **9c** (IC_50_ = 2.4–3.4 µM) clustering at the top, while the coumarin **7a** and thiosemicarbazide **8b** were essentially inactive (IC_50_ ≈ 21–22 µM). Notably, compound **7c** retains low‐micromolar activity against all three enzymes, making it the most balanced member of the series.

#### 2.4.4 Antioxidant Activity

The antioxidant profiles of the synthesized compounds evaluated using HPLC‐based FRAP, DPPH, and CUPRAC assays demonstrated significant variability depending on the molecular structure and assay mechanism.

Among the tested compounds, compound **7c** exhibited the highest overall antioxidant activity, likely due to the presence of the coumarin moiety, which provides an extended conjugated system capable of stabilizing radical intermediates. The ability of coumarin derivatives to act as electron donors is well‐documented and is consistent with their strong performance in both DPPH and FRAP assays.

On the other hand, compound **8a** showed superior activity in the CUPRAC assay, suggesting a strong electron‐transfer capacity toward copper(II) ions. This behavior may be attributed to the presence of thiourea or related functionalities, which can participate in redox processes through sulfur‐centered electron donation.

The differences observed across the three antioxidant assays indicate that the synthesized compounds operate via distinct redox mechanisms, including hydrogen atom transfer (HAT) and single electron transfer (SET). This diversity further supports their potential as multifunctional agents capable of mitigating oxidative stress in neurodegenerative conditions.

Considered together, the coumarin (7‐series) and thiosemicarbazide (8‐series) hybrids drive the redox activity through complementary routes: the coumarin lactone–phenol system of **7c** favors hydrogen‐atom and single‐electron donation, accounting for its dominance in the DPPH and FRAP assays, whereas the sulfur‐rich **8a** excels in the copper‐based CUPRAC assay. The antioxidant response is therefore dictated primarily by the specific electron‐donating group present.

#### 2.4.5 Effect of Liposomal Formulation

The incorporation of selected compounds into liposomal formulations resulted in enhanced antioxidant responses, particularly for compounds **4c, 7c,** and **8a**. This improvement may be attributed to increased solubility, improved dispersion, and enhanced accessibility of the active molecules to reactive species. However, the extent of enhancement varied among compounds, suggesting that the interaction between molecular structure and lipid bilayers plays a critical role in determining the effectiveness of liposomal delivery.

It is noteworthy that the compounds benefiting most from encapsulation (**4c**, **7c,** and **8a**) are also among the more lipophilic members of the series, consistent with efficient partitioning into the phospholipid bilayer, whereas the uneven magnitude of the enhancement points to a structure‐dependent encapsulation efficiency. A quantitative characterization of the liposomes (particle size, polydispersity index, zeta potential, and encapsulation efficiency) would nevertheless be required to firmly establish this structure–delivery relationship.

### 2.5 Molecular Docking Analysis

To predict the binding affinities and evaluate the multi‐target directed ligand potential of the synthesized compounds, docking calculations were performed. The docking scores (kcal/mol) of the target compounds against AChE (PDB ID: 4M0F), BChE (PDB ID: 6QAA), and MAO‐B (PDB ID: 7P4H) are summarized in Table 5, alongside the clinically approved reference drugs Donepezil and Selegiline.

**Table 5:** In silico molecular docking scores (kcal/mol) of the synthesized derivatives and reference drugs.

Compound	AChE	BChE	MAO‐B
**1**	−8.32	−8.03	−7.29
**2**	−12.86	−8.45	−11.58
**3**	−12.64	−8.45	−12.62
**4a**	−10.52	−9.58	−13.83
**4b**	−8.81	−11.62	−10.22
**4c**	−9.33	−10.01	−9.65
**7a**	−9.16	−8.54	−6.25
**7b**	−9.81	−4.70	−7.46
**7c**	−7.93	−7.05	−8.79
**8a**	−6.18	−9.58	−14.00
**8b**	−6.46	−14.20	−5.60
**8c**	−6.93	−8.30	−12.36
**9a**	−9.22	−10.93	−11.74
**9b**	−10.02	−14.00	−14.51
**9c**	−10.06	−10.44	−12.22
**Donepezil**	−16.00	−10.52	
**Selegiline**			−9.22

Among the synthesized series, the most negative AChE docking scores were attained by compounds **2** (−12.86 kcal/mol) and **3** (−12.64 kcal/mol), followed by other elongated derivatives such as **4a** (−10.52 kcal/mol) and **9b** (−10.02 kcal/mol), primarily due to the extensive van der Waals and lipophilic surface contacts generated by their elongated scaffolds across the expanded enzyme gorge. However, it is crucial to highlight the structural performance of compound **1** (−8.32 kcal/mol). Although some bulky derivatives produced numerically higher absolute scores due to the inherent size bias of empirical scoring functions, compound **1** demonstrated an exceptionally optimal binding profile when its highly compact molecular volume is taken into account. This indicates that the core scaffold of compound **1** binds with outstanding ligand efficiency, avoiding severe entropic penalties upon binding, which is consistent with its high in vitro inhibitory potency despite its comparatively modest absolute docking score.

For the BChE (6QAA) enzyme, several bulky derivatives attained more negative docking scores than the reference drug donepezil (−10.52 kcal/mol); this reflects the greater number of contacts available to larger ligands rather than superior potency, as donepezil remained markedly more potent in vitro. Compounds **8b** and **9b** attained the most negative in silico BChE scores within the series (−14.20 and −14.00 kcal/mol, respectively); notably, however, **8b** was among the weakest BChE inhibitors experimentally (IC_50_ = 22.05 µM), illustrating the limited correspondence between docking score and measured potency.

The docking results for MAO‐B provided further evidence for the dual‐target potential of the series. The standard MAO‐B inhibitor selegiline yielded a docking score of −9.22 kcal/mol. Several synthesized hybrids attained more negative MAO‐B docking scores, including **8a** (−14.00 kcal/mol), **9b** (−14.51 kcal/mol), and **9c** (−12.22 kcal/mol); these values should be interpreted as favorable predicted binding geometries rather than superior activity, since selegiline remained far more potent in vitro (IC_50_ = 0.009 µM) and **8a** was only weakly active experimentally (IC_50_ = 8.56 µM).

The comparative docking scores are consistent with the multi‐target engagement of the synthesized series across the three enzymes, although their limited quantitative agreement with the measured potencies is addressed below. To further elucidate the structural basis of these affinities, the specific non‐covalent interactions and spatial orientations of the selected compounds within the enzyme cavities were subjected to detailed visual analysis.

**Figure 9:**
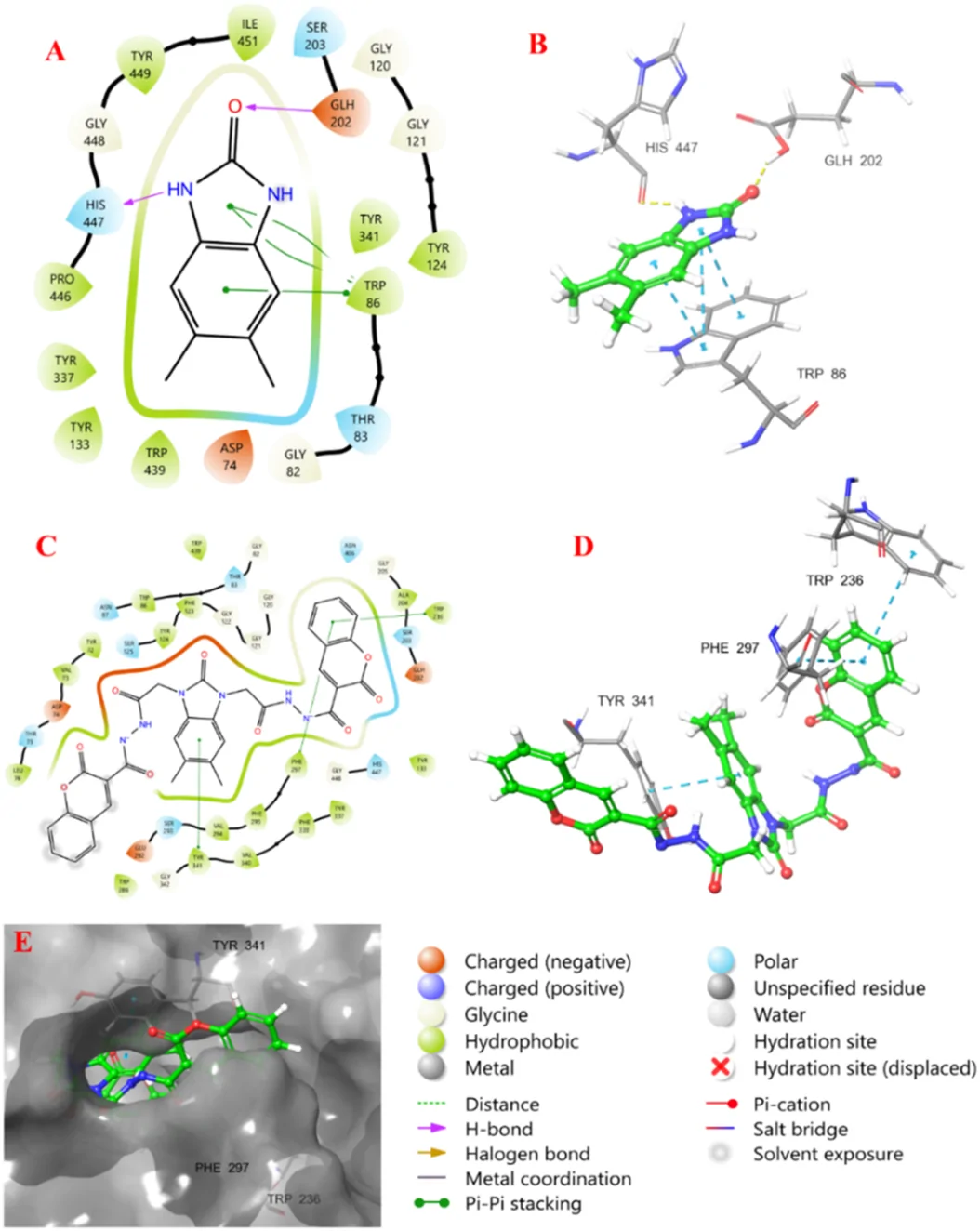
Molecular docking poses and interaction diagrams of Compounds **1** and **7a** within the active site gorge of AChE. (A) 2D ligand interaction diagram of Compound **1**. (B) 3D binding mode of Compound **1** anchored deep within the CAS. (C) 2D ligand interaction diagram of the Compound **7a**. (D) 3D binding mode of Compound **7a**. (E) 3D surface representation of Compound **7a**.

The binding poses of the most active derivative, **1**, and a bulky hybrid derivative, **7a**, on the extended‐gorge conformation of AChE were comparatively analyzed in Figure 9. As depicted in the 2D and 3D interaction diagrams (A and B), Compound **1** precisely localizes deep within the catalytic anionic site (CAS) of the enzyme. Despite its small molecular volume, the benzimidazolone core establishes robust hydrogen bonds with the catalytic triad residues His447 and Glu202. Furthermore, it forms highly stable π–π stacking interactions with Trp86, a critical hallmark of CAS binding. This compact and sterically favorable placement demonstrates that the core scaffold acts as a highly optimized anchor within the deep gorge. The docking poses of the bulky hybrid Compound **7a** (C, D, and E) reveal a significantly extended spatial arrangement. The 3D surface representation (E) clearly illustrates that Compound **7a** spans the entire length of the active site gorge. While the central core is oriented towards the bottom of the CAS gorge, the elongated coumarin moiety simultaneously interacts with the peripheral anionic site (PAS) at the gorge entrance, establishing π‐π stacking with the key peripheral residue Trp286. This extended “Dual‐binding” topology physically occupies both the catalytic and peripheral regions, highlighting how the addition of bulky functional groups drives the structural accommodation of the hybrid ligands across the entire AChE gorge.

**Figure 10:**
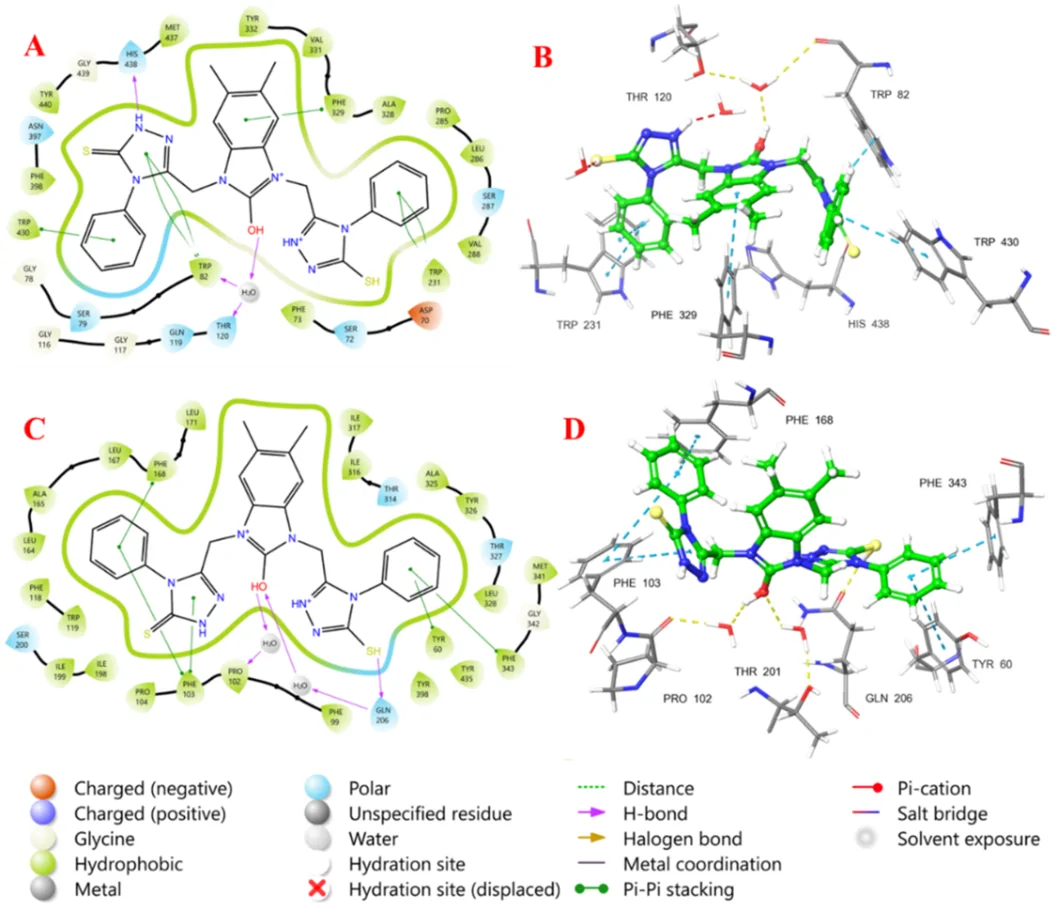
Molecular docking poses demonstrating the multi‐target binding profile of the lead compound, **9b**, within the active sites of BChE and MAO‐B. (A) 2D ligand interaction diagram and (B) 3D binding mode of Compound **9b** within the active site cavity of BChE. (C) 2D ligand interaction diagram and (D) 3D binding mode of Compound **9b** within the active site of MAO‐B.

To further substantiate the multi‐target directed ligand profile of the synthesized series, the binding modes of the most potent dual‐acting derivative, Compound **9b**, were structurally investigated within the BChE and MAO‐B active sites (Figure 10).

Within the BChE (PDB ID: 6QAA) active site, which possesses a naturally more voluminous and flexible acyl pocket compared to AChE, Compound **9b** exhibited an exceptional degree of structural complementarity. As illustrated in the 2D and 3D interaction maps in Figure 10A,B, the bulky hybrid scaffold comfortably accommodates within this expanded cavity without triggering any unfavorable steric clashes. The structural analysis reveals a highly rich network of non‐covalent interactions. Specifically, the terminal aromatic rings and the central core of the molecule establish multiple strong π–π stacking interactions with key residues lining the aromatic gorge, including Trp82, Trp231, Phe329, and Trp430. Furthermore, the presence of critical water‐mediated hydrogen bonds that anchor the molecule to residues such as Thr120 and Trp82 highlights its ability to seamlessly integrate into the native hydration network of the enzyme cavity. This thermodynamically favored spatial arrangement and profound interaction density structurally rationalize its docking score (−14.00 kcal/mol), which is more negative than that of the reference drug donepezil, although donepezil remained substantially more potent against BChE in vitro.

Parallel structural success was observed within the MAO‐B enzyme, providing critical mechanistic insights into its remarkably predicted binding affinity of −14.51 kcal/mol. The MAO‐B active site is characteristically bipartite, featuring an entrance cavity that leads into a deep substrate‐binding pocket, governed by a gating mechanism formed by the Ile199 and Tyr326 residues. As depicted in Figure 10C,D, Compound **9b** successfully exploits this unique and narrow topology. The elongated molecular scaffold effectively traverses the gating region, allowing the molecule to orient its structural moieties to maximize hydrophobic and </w:t > <w:lastRenderedPageBreak/><w:txml:space = “preserve”>electronic contacts across both cavities. The robust interaction profile is primarily driven by vital hydrogen bonding networks and intense π–π stacking interactions, notably with Phe168 and the crucial gating residue Tyr326. By comprehensively occupying this bipartite space and mimicking the spatial trajectory required for potent MAO‐B inhibition, the designed hybrid scaffold demonstrates a favorable predicted binding geometry within the selegiline binding site, notwithstanding the markedly higher in vitro potency of selegiline itself.

The observed discordance between the computed docking scores and the experimental in vitro IC_50_ values is not unexpected and is consistent with broader observations in the docking literature. Comprehensive analyses have repeatedly shown that ΔG values derived from molecular docking do not reliably correlate with experimentally determined binding affinities or potencies; correlation coefficients as low as 0.10–0.38 have been reported across large‐scale comparative studies of multiple docking programs and scoring functions (Pantsar and Poso 2018). Several methodological factors likely contribute to this discrepancy in the present series. First, empirical scoring functions are largely additive and tend to assign more favorable (more negative) scores to larger ligands capable of forming a greater number of individual contacts, which introduces a systematic size bias that can inflate the apparent affinity of the bulkier piperazine‐ and triazole‐bearing hybrids relative to the compact core compound **1** (García‐Ortegón et al. 2022). Second, standard rigid‐receptor docking protocols primarily capture enthalpic contributions such as hydrogen bonding and van der Waals contacts, while largely neglecting the entropic cost of ligand desolvation and the conformational restriction imposed upon binding of larger, more flexible scaffolds—an effect that is expected to penalize extended hybrids more severely under physiological conditions than the scoring function suggests (Pantsar and Poso 2018). Third, docking scores reflect only the intrinsic ligand–receptor interaction energy and do not account for compound‐specific pharmacokinetic factors such as aqueous solubility and free‐fraction availability at the target site; several of the bulkier derivatives in this series (e.g., **4b**, **8b**) were classified as poorly soluble by in silico ADME prediction, which may limit the effective concentration available to the enzyme in the in vitro assay despite a favorable computed binding pose. Taken together, these considerations indicate that the docking scores in this study should be interpreted as a qualitative indicator of plausible binding mode and relative ligand efficiency rather than as a quantitative predictor of in vitro potency, and the strong in vitro activity of the structurally compact compound **1** should not be regarded as inconsistent with its comparatively modest docking score.

### 2.6 In Silico ADME and Drug‐Likeness Prediction

To complement the in vitro and in silico binding studies and to assess the developability of the synthesized hybrids, the physicochemical, pharmacokinetic, and drug‐likeness parameters of all 15 compounds, together with the reference drugs donepezil and selegiline, were predicted using the SwissADME web tool (NEGRU et al. 2025). The results are summarized in Supporting Information S1: Table 7.

The predicted profiles reveal a pronounced dependence of pharmacokinetic behavior on molecular size and polarity. The compact core compound **1** displayed the most favorable drug‐like profile of the series, with high predicted gastrointestinal absorption, positive blood–brain barrier (BBB) permeation, no Lipinski violations, and no structural alerts—a profile comparable to the clinical reference drugs and highly desirable for a centrally‐acting anti‐Alzheimer agent (Ahmad et al. 2024). The piperazine‐bearing hybrids **4a**–**c** also retained high gastrointestinal absorption and predicted BBB permeation owing to their low topological polar surface area (TPSA = 39.67 Å^2^), although their elevated lipophilicity and molecular weight introduced one to two Lipinski violations. In contrast, the larger bis‐conjugated coumarin (**7a–c**), thiosemicarbazide (**8a–c**), and triazole–thiol (**9a–c**) hybrids were uniformly predicted to show low gastrointestinal absorption and no BBB permeation, a direct consequence of their high molecular weight (up to 808 g/mol), high TPSA (up to 289.37 Å^2^), and multiple Lipinski violations; most of these derivatives were also flagged as P‐glycoprotein substrates, which would further limit central nervous system exposure (Maboko et al. 2024). Reassuringly, none of the 15 compounds triggered PAINS alerts, indicating the absence of pan‐assay interference substructures, although the nitro‐ and thiocarbonyl‐containing members (**8a**, **8c**, and **9c**) each raised three Brenk alerts that should be considered in any future optimization. Taken together, the ADME predictions reconcile the potency and docking data with developability considerations: the balance between multi‐target potency and favorable predicted CNS availability is most clearly achieved by the smaller scaffolds, and they identify high molecular weight and polarity as the principal liabilities to be addressed in the further development of the more elaborate hybrids.

## 3 Conclusion

In this study, a new series of benzimidazolone‐based hybrid compounds was successfully designed, synthesized, and biologically evaluated as potential MTDLs for AD. AChE, BChE, and MAO‐B remain among the most extensively pursued pharmacological targets in AD.

Several compounds demonstrated notable inhibitory activity against key enzymes involved in AD pathology. Compound **1** showed the most potent AChE inhibition, compound **9b** exhibited the highest BChE inhibition, and compound **9c** emerged as the most effective MAO‐B inhibitor; taken together, these results indicate multi‐target potential at the series level rather than within a single optimized molecule. All inhibitory activities were determined under standardized experimental conditions using validated colorimetric (Ellman) and fluorometric assay protocols, with triplicate measurements and donepezil and selegiline included as reference inhibitors for the cholinesterase and MAO‐B assays, respectively, ensuring reliable comparability across the compound series.

In addition, the compounds displayed significant antioxidant activity in HPLC‐based FRAP, DPPH, and CUPRAC assays, with compound **7c** showing the highest overall activity. Importantly, liposomal formulations (especially **4c**, **7c**, and **8a**) enhanced antioxidant responses compared to free compounds, suggesting that formulation strategies may further improve their biological performance. These antioxidant responses were quantified as calibration‐curve‐derived equivalent concentrations rather than raw peak areas, allowing direct, concentration‐based comparison of FRAP, DPPH, and CUPRAC activity across the free and liposomal forms of the compound series.

Molecular docking against AChE, BChE, and MAO‐B provided a structural rationale for the observed inhibition, with the active compounds occupying the enzyme gorges through hydrogen bonding and π–π stacking interactions with key catalytic and peripheral residues. It should be noted, however, that the computed docking scores did not correlate quantitatively with the experimental potencies, a limitation that is well documented for empirical scoring functions and that most likely reflects their size bias, the neglect of desolvation and entropic penalties, and compound‐specific solubility differences; the docking results were therefore interpreted as qualitative indicators of binding mode rather than as predictors of potency.

Complementary in silico ADME analysis (SwissADME) showed that favorable drug‐likeness and predicted BBB permeation were confined to the compact scaffold (compound **1**) and the piperazine‐based hybrids (**4a**–**4c**), whereas the larger bis‐conjugated coumarin, thiosemicarbazide, and triazole–thiol derivatives were penalized by high molecular weight, high polar surface area, and multiple Lipinski violations. These predictions highlight the reduction of molecular size and polarity as the key medicinal‐chemistry priority for converting the present multi‐target leads into central‐nervous‐system‐oriented drug candidates.

Overall, these findings indicate that benzimidazolone‐based hybrids represent promising candidates for further development as multifunctional agents against AD.

## 4 Materials and Methods

### 4.1 Chemistry

#### 4.1.1 Synthesis of 5,6‐Dimethyl‐1*H*‐Benzo[d]Imidazol‐2(3*H*)‐One (1)

This compound was synthesized from the reaction of 4,5‐dimethyl‐*o*‐phenylenediamine and urea in diglyme according to our previously published protocol (K. Patil et al. 2023). 4,5‐Dimethyl‐*o*‐phenylenediamine (1.36 g, 10 mmol) and urea (0.90 g, 15 mmol) were dissolved in diglyme (25 mL), and the mixture was boiled for 4 h under a reflux condenser. The mixture was then cooled to room temperature, the precipitate was filtered off, washed with cold acetone, and dried in a vacuum desiccator.

Yield: 1.89 g (98%), m.p.: 320°C–321°C (318°–320°C, Bouchet et al. 1978), ^1^H‐NMR (400 MHz, DMSO‐*d*_*6*_), δ, ppm: 10.33 (s, 2H, 2NH), 6.69 (2H, s, ArH), 2.14 (6H, s, 2CH_3_). ^13^C‐NMR (100 MHz, DMSO‐ *d*_*6*_), δ, ppm: 155.87 (C═O, benzimidazolone), 128.21, 128.18, 110.00 (Ar‐C), 19.85 (CH_3_). Elemental analysis: Calculated, C_9_H_10_N_2_O, C, 66.55; H, 6.21; N, 17.27, found: C, 66.49; H, 6.17; N, 17.22.

#### 4.1.2 Synthesis of Diethyl 2,2′‐(5,6‐Dimethyl‐2‐Oxo‐1*H*‐Benzo[d]Imidazole‐1,3(2*H*)‐Diyl)Diacetate (2)

Compound **1** (1.62 g, 10 mmol) and K_2_CO_3_ (6.90 g, 50 mmol) were added in dry acetone (3 m mL) and stirred at room temperature for 15 min, then ethyl bromoacetate (2.48 mL, 22 mmol) was added and stirred at room temperature overnight. After adding water (100 mL) to the final mixture, the precipitate was filtered off. It was washed with plenty of water, and the resulting product was purified by crystallization in acetone and dried in a desiccator.

Yield: 3.17 g (95%), m.p.: 171°C–173°C, ^1^H‐NMR (400 MHz, DMSO‐ *d*_*6*_), δ, ppm: 6.95 (2H, s, ArH), 4.65 (4H, s, 2NCH_2_), 4.13 (4H, q, *J* = 7.2 Hz, 2OCH_2_), 2.20 (6H, s, 2CH_3_), 1.18 (6H, t, J = 7.2 Hz, 2CH_3_). ^13^C‐NMR (100 MHz, DMSO‐ *d*_*6*_), δ, ppm: 168.52 (C═O, ester), 153.92 (C═O, benzimidazolone), 129.47, 127.50, 109.75 (Ar‐C), 61.51 (OCH_2_), 42.36 (NCH_2_), 19.94 (CH_3_, benzimidazolone 5th and 6th position), 14.47 (CH_3_, ester). Elemental analysis: Calculated, C_17_H_22_N_2_O_5_, C, 61.07; H, 6.63; N, 8.38, found: C, 61.01; H, 6.55; N, 8.32.

#### 4.1.3 Synthesis of 2,2′‐(5,6‐Dimethyl‐2‐Oxo‐1*H*‐Benzo[d]Imidazole‐1,3(2*H*)‐Diyl)Di(Acetohydrazide) (3)

Compound **2** (3.34 g, 10 mmol) was dissolved in ethanol (50 mL). And hydrazine monohydrate (50 mmol) was added to the mixture, and it was boiled for 3 h under reflux conditions. After the reaction was complete, the mixture was cooled to room temperature, and the precipitate was filtered off. The product was purified by washing with ethanol. It was dried in a vacuum desiccator.

Yield: 2.76 g (90%), m.p.: 357°–358°C, ^1^H‐NMR (400 MHz, DMSO‐ *d*_*6*_), δ, ppm: 9.24 (s, 2H, NH), 6.82 (2H, s, ArH), 4.35 (s, 4H, 2NCH_2_), 4.28 (s, 4H, NH_2_), 2.19 (s, 6H, 2CH_3_). ^13^C‐NMR (100 MHz, DMSO‐ *d*_*6*_), δ, ppm: 166.64 (C═O), 154.08 (C═O, benzimidazolone), 129.00, 127.98, 109.60 (Ar‐C), 42.61 (NCH_2_), 19.97 (CH_3_). Elemental analysis: Calculated, C_13_H_18_N_6_O_3_, C, 50.97; H, 5.92; N, 27.44, found: C, 50.91; H, 5.86; N, 27.38.

#### 4.1.4 Synthesis of Compounds 4a–c

Piperazine derivative (22 mmol), DMF (10 mL), and formaldehyde (37%, 2.5 mL) were taken in a reaction balloon and stirred for 10 min at room temperature. Then, compound **1** (1.62 g, 10 mmol) was added, and the mixture was stirred overnight at room temperature. The mixture was poured onto cold water, and the product was precipitated. It was filtered off, washed with water, and dried. It was recrystallized from ethyl acetate to obtain a pure product.

##### 4.1.4.1 5,6‐Dimethyl‐1,3‐Bis((4‐Phenylpiperazin‐1‐yl)Methyl)−1*H*‐Benzo[d]Imidazol‐2(3*H*)‐One (4a)

Yield: 4.43 g (87%), m.p.: 221°–223°C, ^1^H‐NMR (400 MHz, DMSO‐ *d*_*6*_), δ, ppm: 7.16–7.10 (m, 6H, ArH), 6.91–6.82 (m, 4H, ArH), 6.72 (m, 2H, ArH), 4.64 (s, 4H, 2NCH_2_), 3.07 (brs, 8H, piperazine CH_2_), 2.69 (brs, 8H, piperazine CH_2_), 2.17 (s, 6H, 2CH_3_). ^13^C‐NMR (100 MHz, DMSO‐*d*_*6*_), δ, ppm: 155.50 (C═O, benzimidazolone), 151.41, 129.33, 129.26, 128.38, 119.28, 115.99, 110.31 (Ar‐C), 62.52 (NCH_2_), 50.50, 48.62 (piperazine CH_2_), 20.07 (CH_3_). Elemental analysis: Calculated, C_31_H_38_N_6_O, C, 72.91; H, 7.50; N, 16.46, found: C, 72.84; H, 7.46; N, 16.42.

##### 4.1.4.2 1,3‐Bis((4‐(4‐Chlorophenyl)Piperazin‐1‐yl)Methyl)−5,6‐Dimethyl‐1*H*‐Benzo[d]Imidazol‐2(3*H*)‐One (4b)

Yield: 4.71 g (85%), m.p.: 270°–272°C, ^1^H‐NMR (400 MHz, DMSO‐*d*_*6*_), δ, ppm: 7.17 (m, 6H, ArH), 6.88 (m, 4H, ArH), 4.63 (s, 4H, 2NCH_2_), 3.08 (brs, 8H, piperazine CH_2_), 2.66 (brs, 8H, piperazine CH_2_), 2.21 (s, 6H, 2CH_3_). ^13^C‐NMR (100 MHz, DMSO‐*d*_*6*_), δ, ppm: 155.49 (C═O, benzimidazolone), 151.40, 129.71, 129.26, 129.68, 129.28, 115.97, 110.31 (Ar‐C), 62.52 (NCH_2_), 50.51, 48.62 (piperazine NCH_2_), 20.07 (CH_3_). Elemental analysis: Calculated, C_31_H_36_Cl_2_N_6_O, C, 64.24; H, 6.26; N, 14.50, found: C, 64.19; H, 6.21; N, 14.45.

##### 4.1.4.3 1,3‐Bis((4‐(4‐Fluorobenzyl)Piperazin‐1‐yl)Methyl)−5,6‐Dimethyl‐1*H*‐Benzo[d]Imidazol‐2(3*H*)‐One (4c)

Yield: 4.93 g (84%), m.p.: 159°–160°C, ^1^H‐NMR (400 MHz, DMSO‐ *d*_*6*_), δ, ppm: 7.27–7.21 (m, 4H, ArH), 7.09–6.98 (m, 6H, ArH), 4.55 (s, 4H, 2NCH_2_), 3.37 (s, 4H, CH_2_), 2.52 (brs, 8H, piperazine CH_2_), 2.30 (brs, 8H, piperazine CH_2_), 2.19 (s, 6H, 2CH_3_). ^13^C‐NMR (100 MHz, DMSO‐*d*_*6*_), δ, ppm: 161.60 (C_F_, *J* = 240.8 Hz), 155.45 (C═O, benzimidazolone), 134.79, 130.96, 130.88, 129.36, 115.34, 110.26 (Ar‐C), 62.49 (NCH_2_), 61.38 (CH_2_), 52.70, 50.47 (NCH_2_), 20.00 (CH_3_). Elemental analysis: Calculated, C_33_H_40_F_2_N_6_O, C, 68.97; H, 7.02; N, 14.62, found: C, 68.91; H, 9.96; N, 14.58.

#### 4.1.5 Synthesis of Compounds 5a–c

A mixture of corresponding salicyl aldehyde (10 mmol) and 2,2‐dimethyl‐1,3‐dioxane‐4,6‐dione (Meldrum's acid) (1.58 g, 11 mmol) in ethanol (50 mL, containing 0.5 ml of pyridine) was refluxed in a round‐bottom flask for 6 h. After completion of the reaction (monitored by TLC, ethyl acetate:hexane, 4:1), the solvent was evaporated under reduced pressure. The residue was washed with water and recrystallized from ethanol:water (3:2).

##### 4.1.5.1 2‐Oxo‐2 H‐Chromene‐3‐Carboxylic Acid (5a)

Yield: 1.39 g (73%), m.p.: 189°C–190°C (189°–190°C, Yilmaz et al. 2021).

##### 4.1.5.2 6‐Chloro‐2‐Oxo‐2 H‐Chromene‐3‐Carboxylic Acid (5b)

Yield: 2.20 g (63%), m.p.: 194°–195°C (194°–195°C, Yilmaz et al. 2021).

##### 4.1.5.3 6‐Bromo‐2‐Oxo‐2 H‐Chromene‐3‐Carboxylic Acid (5c)

Yield: 1.80 g (67%). m.p.: 197°C–198°C (195°C–196°C, Yilmaz et al. 2021).

#### 4.1.6 Synthesis of Compounds 6a–c

Compounds **5a–c** (10 mmol) were dissolved in dichloromethane (75 mL), and thionyl chloride (1.78 g, 15 mmol) was added. The mixture was stirred for 30 min at room temperature, and 1H‐benzotriazole (5.95 g, 50 mmol) was added. Then, the mixture was stirred overnight at room temperature. The precipitated salt was filtered off, and the filtrate was washed with 10% Na_2_CO_3_ solution (50 mL) and 1 N HCl (50 mL). Then, the solvent was removed under reduced pressure, and the product was obtained as a white solid. It was recrystallized from Dichloromethane/hexane, 1/1.

##### 4.1.6.1 3‐(1H‐Benzotriazol‐1‐Ylcarbonyl)−2H‐Chromen‐2‐One (6a)

Yield: 2.12 g (73%), m.p.: 180°C–181°C (179°C–180°C, Yilmaz et al. 2021).

##### 4.1.6.2 3‐(1H‐Benzotriazol‐1‐Ylcarbonyl)−6‐Chloro‐2H‐Chromen‐2‐One (6B)

Yield: 2.20 g (63%), m.p.: 248°C–249°C (mp 248°C–249°C, Yilmaz et al. 2021).

##### 4.1.6.3 3‐(1H‐Benzotriazol‐1‐Ylcarbonyl)−6‐Bromo‐2H‐Chromen‐2‐One (6c)

Yield: 2.52 g (68%), m.p.: 250°C–251°C (250°C–251°C, Yilmaz et al. 2021).

#### 4.1.7 Synthesis of Compounds 7a–c

Compound **3** (3.07 g, 10 mmol) was dissolved in DMSO (5 mL), and the mixture was stirred for 10 min at room temperature. Then, the corresponding compound **6a–c** (22 mmol) was added. Then the mixture was heated at 80°C for 7 h under reflux conditions. The final mixture was poured onto the ice‐water mixture, and the precipitated substance was filtered off and washed with water and hot ethanol for purification.

##### 4.1.7.1 *N′,N‴*‐(2,2′‐(5,6‐Dimethyl‐2‐Oxo‐1*H*‐Benzo[d]Imidazole‐1,3(2H)‐Diyl)Bis(Acetyl))Bis(2‐Oxo‐2*H*‐Chromene‐3‐Carbohydrazide) (7a)

Yield: 5.07 g (72%), m.p.: 348°–349°C, ^1^H‐NMR (400 MHz, DMSO‐*d*_*6*_), δ, ppm: 11.13 (s, 1H, NH), 10.63 (s, 2H, NH), 8.87 (s, 2H, ArH, coumarin C_3_H), 7.99 (d, *J* = 6.8 Hz, 2H, ArH), 7.74 (m, 2H, ArH), 7.48 (m, 4H, ArH), 6.94 (s, 2H, ArH), 4.61 (s, 4H, NCH2), 2.21 (s, 6H, 2CH_3_). ^13^C‐NMR (100 MHz, DMSO‐d6), δ, ppm: 164.52, 160.22, 159.26 (C═O), 154.37 (coumarin C_3_), 153.92 (C═O, benzimidazolone), 148.44 (coumarin C_4_H), 134.87, 130.80, 129.88, 129.85, 125.67, 118.71, 116.68, 110.72, 109.99 (Ar‐C), 42.41 (NCH_2_), 19.04 (CH_3_). Elemental analysis: Calculated, C_33_H_26_N_6_O_9_, C, 60.92, H, 4.03, N, 12.92; found: C, 60.86, H, 3.97, N, 12.88.

##### 4.1.7.2 *N′,N‴*‐(2,2′‐(5,6‐Dimethyl‐2‐Oxo‐1*H*‐Benzo[d]Imidazole‐1,3(2*H*)‐Diyl)Bis(Acetyl))Bis(6‐Chloro‐2‐Oxo‐2*H*‐Chromene‐3‐Carbohydrazide) (7b)

Yield: 5.11 g (70%), m.p.: 355°–357°C, ^1^H‐NMR (400 MHz, DMSO‐*d*_*6*_), δ, ppm: 11.22 (s, 1H, NH), 10.61 (s, 2H, NH), 8.82 (s, 2H, ArH, coumarin C_3_H), 8.13 (s, 2H, ArH), 7.78 (d, *J* = 8.4 Hz, 2H, ArH), 7.54 (d, *J* = 6.8 Hz, 2H, ArH), 6.94 (s, 2H, ArH), 4.60 (s, 4H, NCH_2_), 2.22 (s, 6H, 2CH_3_). ^13^C‐NMR (100 MHz, DMSO‐d6), δ, ppm: 165.05, 159.76, 159.06, 154.14 (C═O), 153.00 (coumarin C_3_), 147.05 (coumarin C_4_H), 134.20, 129.58, 129.31, 129.22, 127.85, 120.10, 119.82, 109.89 (Ar‐C), 42.18 (NCH_2_), 20.00 (CH_3_). Elemental analysis: Calculated, C_33_H_24_Cl_2_N_6_O_9_, C, 55.09, H, 3.36, N, 11.68; found: C, 55.02, H, 3.33, N, 11.64.

##### 4.1.7.3 *N′,N‴*‐(2,2′‐(5,6‐Dimethyl‐2‐Oxo‐1*H*‐Benzo[d]Imidazole‐1,3(2*H*)‐Diyl)Bis(Acetyl))Bis(6‐Bromo‐2‐Oxo‐2*H*‐Chromene‐3‐Carbohydrazide) (7c)

Yield: 5.50 g (68%), m.p.: 370°–372°C, ^1^H‐NMR (400 MHz, DMSO‐*d*_*6*_), δ, ppm: 11.11 (s, 1H, NH), 10.80 (s, 2H, NH), 8.81 (s, 2H, ArH, coumarin C_3_H), 8.24 (s, 2H, ArH), 7.88 (d, *J* = 7.4 Hz, 2H, ArH), 7.47 (d, *J* = 6.8 Hz, 2H, ArH), 6.93 (s, 2H, ArH), 4.60 (s, 4H, NCH_2_), 2.21 (s, 6H, 2CH_3_). ^13^C‐NMR (100 MHz, DMSO‐d6), δ, ppm: 165.06, 159.72, 159.07, 154.14 (C═O), 153.41 (coumarin C_3_), 146.98 (coumarin C_4_H), 136.95, 132.56, 129.22, 127.85, 120.59, 119.78, 118.97, 117.15, 109.87 (Ar‐C), 42.19 (NCH_2_), 19.99 (CH_3_). Elemental analysis: Calculated, C_33_H_24_Br_2_N_6_O_9_, C, 49.03, H, 2.99, N, 10.40; found: C, 48.95, H, 2.94, N, 10.37.

#### 4.1.8 Synthesis of Compounds 8a–c

A mixture of compounds **3** (10 mmol) and corresponding isothiocyanate derivatives (22 mmol) was refluxed in ethanol (10 mL) for 3 h. The solution was poured into water, and the precipitated substance was filtered, washed with plenty of water, and recrystallized from ethanol to afford the pure product.

##### 4.1.8.1 2,2′‐(2,2′‐(5,6‐Dimethyl‐2‐Oxo‐1*H*‐Benzo[d]Imidazole‐1,3(2*H*)‐Diyl)Bis(Acetyl))Bis(N‐Ethylhydrazinecarbothioamide) (8a)

Yield: 4.37 g (91%), m.p.: 222°–224°C, ^1^H‐NMR (400 MHz, DMSO‐*d*_*6*_), δ, ppm: 10.11 (s, 2H, NH), 9.26 (s, 2H, NH), 8.00 (s, 2H, NH), 6.89 (s, 2H, ArH), 4.51 (s, 4H, NCH2), 3.48 (q, *J* = 6.4 Hz,4H, 2CH_2_), 2.21 (s, 6H, 2CH_3_), 1.07 (t, *J* = 6.4 Hz,6H, 2CH_3_). ^13^C‐NMR (100 MHz, DMSO‐d6), δ, ppm: 186.72 (C═S), 167.01 (C═O), 154.26 (C═O, benzimidazolone), 129.23, 127.70, 109.97 (Ar‐C), 42.94 (NCH_2_), 38.95 (CH_2_), 19.93 (CH_3_, 5th and 6th benzimidazolone), 14.88 (CH_3_). Elemental analysis: Calculated, C_19_H_28_N_8_O_3_S_2_, C, 47.48, H, 5.87, N, 23.31; found: C, 47.42, H, 5.83, N, 23.26.

##### 4.1.8.2 2,2′‐(2,2′‐(5,6‐Dimethyl‐2‐Oxo‐1*H*‐Benzo[d]Imidazole‐1,3(2*H*)‐Diyl)Bis(Acetyl))Bis(N‐Phenylhydrazinecarbothioamide) (8b)

Yield: 5.47 g (95%), m.p.: 258°–260°C, ^1^H‐NMR (400 MHz, DMSO‐*d*_*6*_), δ, ppm: 10.40 (s, 2H, NH), 9.67 (s, 2H, NH), 9.28 (s, 2H, NH), 7.44 (m, 4H, ArH), 7.33 (t, *J* = 7.6 Hz, 4H, ArH), 7.18 (t, *J* = 7.6 Hz, 2H, ArH) 6.93 (s, 2H, ArH), 4.59 (s, 4H, NCH2), 2.20 (s, 6H, 2CH_3_).^13^C‐NMR (100 MHz, DMSO‐*d*_*6*_), δ, ppm: 166.66 (C═O), 154.35 (C═O, benzimidazolone), 139.42, 129.30, 129.23, 129.02, 128.53, 127.93, 127.73, 114.73, 109.61 (Ar‐C), 42.62 (NCH_2_), 19.97 (CH_3_). Elemental analysis: Calculated, C_27_H_28_N_8_O_3_S_2_, C, 56.23, H, 4.89, N, 19.43; found: C, 56.17, H, 4.84, N, 19.40.

##### 4.1.8.3 2,2′‐(2,2′‐(5,6‐Dimethyl‐2‐Oxo‐1H‐Benzo[d]Imidazole‐1,3(2H)‐Diyl)Bis(Acetyl))Bis(N‐(4‐Nitrophenyl)Hydrazinecarbothioamide) (8c)

Yield: 5.46 g (82%), m.p.: 214°–215°C, ^1^H‐NMR (400 MHz, DMSO‐*d*_*6*_), δ, ppm: 10.53 (s, 2H, NH), 10.67 (s, 2H, NH), 9.45 (s, 2H, NH), 8.21 (d, *J* = 7.6 Hz, 4H, ArH), 7.93 (d, *J* = 7.6 Hz, 4H, ArH) 6.96 (s, 2H, ArH), 4.61 (s, 4H, NCH2), 2.21 (s, 6H, 2CH_3_).^13^C‐NMR (100 MHz, DMSO‐*d*_*6*_), δ, ppm: 180.77 (C═S), 156.12 (C═O), 154.36 (C═O, benzimidazolone), 145.87, 129.43, 127.87, 126.82, 124.25, 112.80 (Ar‐C), 42.75 (NCH_2_), 19.95 (CH_3_). Elemental analysis: Calculated, C_27_H_26_N_10_O_7_S_2_, C, 48.64, H, 3.93, N, 21.01; found: C, 48.57, H, 3.86, N, 20.93.

#### 4.1.9 Synthesis of Compounds 9a–c

A solution of corresponding thiosemicarbazide derivative (**8a–c**) (1 mmol) in 2 N NaOH (aq) (for **9a,b**) or 2 N NaHCO_3_ (for **9c**) (10 mL) was refluxed for 12 h. After cooling to room temperature, the reaction mixture was acidified to pH 5 with 37% HCl. The precipitated solid was filtered, washed with water, dried, and recrystallized from ethanol to obtain the pure products.

##### 4.1.9.1 1,3‐Bis((4‐Ethyl‐5‐Mercapto‐4*H*−1,2,4‐Triazol‐3‐yl)Methyl)−5,6‐Dimethyl‐1*H*‐Benzo[d]Imidazol‐2(3H)‐One (9a)

Yield: 3.60 g (81%), m.p.: 348°–350°C, ^1^H‐NMR (400 MHz, DMSO‐*d*_*6*_), δ, ppm: 13.86 (s, 2H, SH), 6.98 (s, 2H, ArH), 5.20 (s, 4H, NCH2), 4.20 (q, *J* = 6.8 Hz, 4H, CH_2_), 2.17 (s, 6H, 2CH_3_), 1.08 (t, *J* = 6.8 Hz, 4H, CH_3_). ^13^C‐NMR (100 MHz, DMSO‐*d*_*6*_), δ, ppm: 167.39 (C═N), 153.31 (C═O, benzimidazolone), 148.01 (C═N), 130.06, 127.06, 110.21 (Ar‐C), 39.00 (NCH_2_), 36.30 (CH_2_), 20.05 (CH_3_), 13.58 (CH_3_). Elemental analysis: Calculated, C_19_H_24_N_8_OS_2_, C, 51.33, H, 5.44, N, 25.20; found: C, 51.28, H, 5.40, N, 25.15.

##### 4.1.9.2 1,3‐Bis((5‐Mercapto‐4‐Phenyl‐4*H*−1,2,4‐Triazol‐3‐yl)Methyl)−5,6‐Dimethyl‐1H‐Benzo[d]Imidazol‐2(3*H*)‐One (9b)

Yield: 4.10 g (76%), m.p.: 330°–332°C, ^1^H‐NMR (400 MHz, DMSO‐*d*_*6*_), δ, ppm: 13.89 (s, 2H, SH), 7.44 (d, *J* = 5.6 Hz, 6H, ArH), 7.27 (t, *J* = 5.6 Hz, 4H, ArH), 6.70 (s, 2H, ArH), 4.77 (s, 4H, NCH2), 2.16 (s, 6H, 2CH_3_). ^13^C‐NMR (100 MHz, DMSO‐*d*_*6*_),δ, ppm: 168.86 (C═N), 152.49 (C═O, benzimidazolone), 148.07 (C═N), 132.27, 129.95, 129.80, 129.50, 128.29, 126.87, 109.23 (Ar‐C), 36.56 (NCH_2_), 19.99 (CH_3_). Elemental analysis: Calculated, C_27_H_24_N_8_OS_2_, C, 59.98; H, 4.47; N, 20.73, found: C, 59.93; H, 4.43; N, 20.68.

##### 4.1.9.3 1,3‐Bis((5‐Mercapto‐4‐(4‐Nitrophenyl)−4H‐1,2,4‐Triazol‐3‐yl)Methyl)−5,6‐Dimethyl‐1H‐Benzo[d]Imidazol‐2(3H)‐One (9c)

Yield: 4.22 g (67%), m.p.: 207°–209°C, ^1^H‐NMR (400 MHz, DMSO‐*d*_*6*_), δ, ppm: 14.04 (s, 2H, SH), 8.17 (d, *J* = 8.8 Hz, 4H, ArH), 7.55 (d, *J* = 8.8 Hz, 4H, ArH), 6.58 (s, 2H, ArH), 4.86 (s, 4H, NCH2), 2.05 (s, 6H, 2CH_3_). ^13^C‐NMR (100 MHz, DMSO‐*d*_*6*_),δ, ppm: 168.71 (C═N), 152.37 (C═O, benzimidazolone), 148.00 (C═N), 147.94, 138.68, 130.03, 129.33, 126.50, 125.01, 109.65 (Ar‐C), 36.72 (NCH_2_), 19.66 (CH_3_). Elemental analysis: Calculated, C_27_H_22_N_10_O_5_S_2_, C, 51.42; H, 3.52; N, 22.21, found: C, 51.37; H, 3.48; N, 22.18.

### 4.2 Biological Activity

#### 4.2.1 In Vitro Cholin‐Ergic Enzymes (AChE and BChE) Inhibition Studies

All the newly synthesized compounds were evaluated to cholin‐ergic enzymes (AChE and BChE) inhibition activity, according to Ellman's colorimetric method (Ellman et al. 1961). AChE (from Electrophorus electricus, Type VI‐S; Sigma‐Aldrich, C2888) and BChE (from equine serum; Sigma‐Aldrich, C7512) enzyme solutions were prepared in 1% gelatin solution as 2.5 units/mL concentration. AChE or BChE (50 μL), different concentrations of the tested compounds (50 μL), and ATC (20 μL, 75 mM) or butrylthiocholine chloride (BTC, 20 μL, 75 mM) as substrate, were added to 1500 µL phosphate buffer (pH 8.0, 0.1 M) and incubated at 25°C for 20 min. After the process had been initiated, chromatographic reagent 5,5‐dithio‐bis(2‐nitrobenzoic) acid (DTNB, 100 μL, 10 mM) was added to the enzyme‐inhibitor mixture. After 20 min at 37°C of yellow anion generation, a UV/vis spectrophotometer was used to determine the absorbance at 412 nm. It was determined by preparing an identical solution of the enzyme in the absence of the tested compounds (as a control). All operations were repeated three times. The IC_50_ value was determined by linear regression analysis between percent inhibition and sample concentration using the Excel program. Donepezil hydrochloride was used as a standard, and results were compared. The cholinesterase inhibition percentage was calculated as follows:

AChE or BChEinhibition⁡(%)=[(Acontrol−Asample)/Acontrol]×100

In the reaction media, the different substrate (ATC or BTC) concentrations were 0.25, 0.5, 0.75, 1.0, and 1.5 mM in the buffer (pH 8.0) containing enzyme solution, with inhibitor or without inhibitor solutions. To obtain information about the inhibition type of these compounds on the AChE and BChE enzymes, the Lineweaver–Burke graphs were obtained, *K*_m_ and *V*_max_ values were calculated, and inhibition patterns were evaluated.

#### 4.2.2 MAO‐B Inhibitory Assay

MAO‐B inhibition was evaluated in 96‐well black plates using recombinant human MAO‐B (Sigma‐Aldrich, M7441) and a continuous HRP‐coupled Amplex/Resorufin readout. Test compounds (15 total) and the reference inhibitor were prepared in 2% DMSO over a 10^−3^–10^−9^ M range; after pre‐incubating 20 µL inhibitor with 100 µL enzyme at 37°C for 30 min, the reaction was initiated by adding 100 µL working solution comprising HRP, Amplex (Ampliflu Red), and p‐tyramine, and fluorescence was recorded kinetically at ~Ex/Em 560/590 nm over 30 min. Background (enzyme‐free) signals were subtracted, percent inhibition was calculated, and IC_50_ values were obtained by nonlinear regression (log [inhibitor] vs. response, variable slope) in GraphPad Prism. The choice of recombinant hMAO‐B as the enzyme source is consistent with established practices for reliable inhibitor screening and comparability to platelet MAO‐B (Novaroli et al. 2005).

### 4.3 On‐Line HPLC Methodologies

#### 4.3.1 HPLC Analysis

Analyses were performed on an Agilent 1100 modular HPLC equipped with autosampler, UV‑DAD, a thermostatted column compartment, and a post‑column reagent line driven by a secondary syringe pump for on‑line antioxidant detection (FRAP/DPPH/CUPRAC). Separations used an encapped Purospher Star RP‑18 column with guard (5 µm, 4.6 × 250 mm; Merck). Injection volume was 20 µL and total run‑time 30 min. Data processing (retention times, peak areas, LOD/LOQ) was carried out in Agilent ChemStation. The “Concentration (ppm)” values, reported in Supporting Information S1: Tables 1–6, correspond to equivalent concentrations calculated from external calibration curves constructed for each detection channel (280, 595, 517, and 450 nm), rather than raw peak‐area values. Mobile phases were quaternary: A = MeOH; B = formic acid/ACN/water (3.5/48.25/48.25, v/v/v); C = 1.5% formic acid in water (v/v); D = ACN. A compact gradient was applied: 0–15 min, 10%–35% A/30%–65% B; 15–18 min, 65%–80% A/15%–20% C; 18–20 min, 20%–70% A/0%–10% B/0%–20% C; 20–22 min, 20% A/80% D; 22–25 min, 10%–15% A/0%–70% B/0%–15% D; 25–30 min, re‑equilibration at 15% A/85% B. Post‑column derivatization and detection followed established on‑line HPLC antioxidant schemes: CUPRAC at 450 nm, DPPH at 517 nm, and FRAP at ~595 nm, enabling peak‑specific antioxidant responses alongside UV‑DAD chromatograms (Arslan Burnaz et al. 2017).

#### 4.3.2 HPLC‐FRAP Methodology

An online HPLC–FRAP post‐column antioxidant detection system was employed to profile 15 synthesized compounds and their liposomal formulations under identical chromatographic conditions. The main pump was set to 0.70 mL min^−1^; UV‐DAD acquired analytical chromatograms at 280 nm, while the FRAP channel monitored the ~595 nm response. A secondary reagent pump delivered freshly prepared FRAP reagent through a PTFE reaction coil (0.22 mm i.d., 1.5 m) in parallel flow; the column oven was maintained at 23°C–25°C. Reagents and samples were 0.20 µm filtered and each sample (free and liposomal) was analyzed in triplicate. Method linearity was verified within the study's working range; LOD/LOQ were estimated from noise (3× and 10× SD) per channel, and all values were computed in ChemStation. This configuration follows the classical FRAP chemistry for electron‐transfer‐based antioxidant readout in flow, adapted here to yield peak‐specific FRAP responses for both free compounds and their liposomal forms (Benzie and Strain 1996).

#### 4.3.3 HPLC‐DPPH Methodology

An online HPLC–DPPH post‐column scavenging assay (negative detection at 517 nm) was applied to 15 synthesized compounds and their liposomal formulations, using exactly the same main/secondary pump flow rates, column temperature, solvent gradient, and 20 µL injection volume as in the HPLC–FRAP setup. Fresh DPPH radical reagent was delivered by the secondary pump to a post‐column reaction coil operated in parallel flow; chromatographic separation and peak quantitation were recorded by UV‐DAD at 280 nm, while DPPH quenching signals were monitored simultaneously at 517 nm. Each free‐compound and liposomal sample was analyzed in triplicate. LOD/LOQ were estimated from baseline noise (3× and 10× SD, respectively) for both 280 and 517 nm channels, and results were processed in ChemStation. Method linearity was confirmed across the study's working range under these conditions. This configuration follows established on‐line HPLC–DPPH post‐column methodology for peak‐specific assessment of radical‐scavenging activity during chromatographic separation (Ou et al. 2013).

#### 4.3.4 HPLC‐CUPRAC Methodology

An online HPLC–CUPRAC post‐column assay (negative detection at 450 nm) was employed to quantify peak‐specific antioxidant responses for 15 synthesized compounds and their liposomal formulations. The method used exactly the same main/secondary pump flow rates, column temperature, solvent gradient, and 20 µL injection volume as the HPLC–FRAP setup. A freshly prepared Cu(II)–neocuproine reagent stream was delivered by the secondary pump into a PTFE reaction coil operated in parallel flow; chromatographic separation and quantification were recorded by UV‐DAD at 280 nm, while CUPRAC quenching signals were monitored simultaneously at 450 nm. Each free‐compound and liposomal sample was analyzed in triplicate. LOD/LOQ were estimated from baseline noise (3× and **10× SD, respectively) for both 280 and 450 nm channels, with calculations performed in ChemStation. This configuration follows the established on‐line HPLC–CUPRAC post‐column approach for peak‐resolved antioxidant screening during separation (Çelik et al. 2010). All figures and chromatograms have been replaced with high‐resolution versions to improve clarity and readability.

## 5 Molecular Docking Studies

### 5.1 Protein and Ligand Preparation

In silico molecular docking studies were performed using the Schrödinger Suite 2018‐4 software package (Schrödinger Release 2018‐4, Maestro, Schrödinger LLC, New York, NY 2018). To elucidate the binding modes of the synthesized hybrid compounds at the molecular level, three‐dimensional high‐resolution crystal structures of the target enzymes were retrieved from the Protein Data Bank. For AChE, PDB ID: 4M0F (2.30 Å resolution) co‐crystallized with Territrem B was selected to properly accommodate the bulky dual‐binding ligands (Cheung et al. 2013). For BChE and MAO‐B, the crystal structures with PDB ID: 6QAA (1.90 Å resolution) and 7P4H (2.10 Å resolution) were utilized, respectively (Ekström et al. 2022; Meden et al. 2019). The selection of the 7P4H structure for MAO‐B is particularly well‐suited for this study, as it represents the enzyme co‐crystallized with a coumarin‐bearing dual inhibitor, providing an ideal template for evaluating structurally related multi‐target directed ligands.

The protein structures were refined using the Protein Preparation Wizard module (Madhavi Sastry et al. 2013). The preparation process included assigning bond orders, adding hydrogen atoms, creating zero‐order bonds to metals, and filling missing side chains using Prime (Jacobson et al. 2004). The protonation and tautomeric states of the amino acid residues at physiological pH (7.4 ± 0.2) were optimized using the PROPKA tool. Finally, restrained minimization of the protein structures was carried out using the OPLS4 force field until the root‐mean‐square deviation (RMSD) of the heavy atoms converged to 0.30 Å.

The 3D structures of the synthesized ligands and the reference drugs (Donepezil and Selegiline) were generated and geometry‐optimized using the LigPrep module (Schrödinger Release 2018‐4: LigPrep, Schrödinger LLC, New York, NY 2018). Possible protonation states at pH 7.4 ± 2.0 were assigned using Epik, and the geometries were minimized using the OPLS4 force field to generate the lowest‐energy conformers for docking.

### 5.2 Docking Protocol

To account for receptor flexibility and accurately capture the structural adaptations during ligand binding, an induced fit docking (IFD) protocol was performed utilizing the Schrödinger Suite (Sherman et al. 2006). The docking site was defined based on the centroid of the co‐crystallized native ligand for each target enzyme.

During the initial docking phase, a softened potential with a van der Waals radii scaling of 0.50 for both the protein and ligand atoms was applied to facilitate the accommodation of the bulky hybrid scaffolds into the active site cavities and the surrounding peripheral subsites. Following the initial ligand placement, residue side chains within a 5.0 Å radius of the docked poses were refined using the Prime module to optimize localized structural complementarity and resolve any steric clashes. In the final stage, the ligands were rigorously re‐docked into the newly optimized, flexible receptor conformations using the Glide Extra Precision (XP) mode (Friesner et al. 2006). Binding affinities were quantified via DockingScore (kcal/mol), and the non‐covalent interactions of the top‐ranked poses were subsequently visualized and analyzed to elucidate the binding mechanisms.

### 5.3 Validation of the Docking Protocol

To validate the reliability and predictive accuracy of the employed IFD‐Glide XP docking protocol, a redocking procedure was conducted. The native co‐crystallized ligands, Territrem B for AChE, the tryptophane‐based derivative for BChE, and the reference inhibitor for MAO‐B, were extracted from their respective PDB structures and subsequently re‐docked into the receptor binding sites using the exact IFD parameters described above. The validity of the methodology was confirmed by calculating the RMSD between the experimentally determined crystal poses and the generated docking poses. In all cases, the obtained RMSD values were found to be below 2.0 Å, indicating that the chosen protocol successfully reproduces the native binding geometries and is robust enough for the predictive evaluation of the synthesized novel hybrid derivatives.

## Author Contributions

**Fatih Yılmaz:** investigation, writing – review and editing, methodology, conceptualization, and formal analysis. **Emre Menteşe:** project administration, methodology. **Ozan Emre Eyüpoğlu:** methodology, investigation, writing – review and editing. **Bahittin Kahveci:** methodology, investigation. **Mustafa Emirik:** methodology, investigation, writing – review and editing.

## Ethics Statement

The authors have nothing to report.

## Conflicts of Interest

The authors declare no conflicts of interest.

## Supporting information


Supporting File 1

Supporting File 2

## Data Availability

The data that support the findings of this study are available in the Supporting Material of this article.
