# Supplementary material for: Design, Synthesis, and Biological Evaluation of Benzimidazol‐2‐One Hybrids as Potential Anti‐Alzheimer's Disease Agents
Source: Drug Dev Res. 2026 Aug 20;87(6):e70369. doi: 10.1002/ddr.70369 (PMC13492513; doi:10.1002/ddr.70369)
Supplement: Supplementary file 1 [file DDR-87-e70369-s001.docx]

**Suplementary Material**

**Design, Synthesis and Biological Evaluation of Benzimidazol-2-one Hybrids as Potential anti-Alzheimer’s Disease Agents**

Fatih Yılmaz^1^, Emre Menteşe^2^*, Ozan Emre Eyüpoğlu^3^, Bahittin Kahveci^4^

*^1^Vocational School of Technical Sciences, Department of Chemistry and Chemical Process Technology, Recep Tayyip Erdogan University, Rize, Turkey*

*^2^Department of Chemistry, Faculty of Art and Sciences, Recep Tayyip Erdogan University, Rize, Turkey*

*^3^Department of Biochemistry, School of Pharmacy, Istanbul Medipol University, Istanbul, Turkey*

*^4^Department of Nutrition and Dietetics, Faculty of Health Sciences, Karadeniz Technical University, Trabzon, Turkey*

*corresponding author e-mail: emre.mentese@erdogan.edu.tr

^1^H NMR spectra of compound **1**

^13^C NMR spectra of compound **1**

^1^H NMR spectra of compound **2**

^13^C NMR spectra of compound **2**

^1^H NMR spectra of compound **3**

^13^C NMR spectra of compound **3**

^1^H NMR spectra of compound **4a**

^13^C NMR spectra of compound **4a**

^1^H NMR spectra of compound **4b**

^13^C NMR spectra of compound **4b**

^1^H NMR spectra of compound **4c**

^13^C NMR spectra of compound **4c**

^1^H NMR spectra of compound **7a**

^13^C NMR spectra of compound **7a**

^1^H NMR spectra of compound **7b**

^13^C NMR spectra of compound **7b**

^1^H NMR spectra of compound **7c**

^13^C NMR spectra of compound **7c**

^1^H NMR spectra of compound **8a**

^13^C NMR spectra of compound **8a**

^1^H NMR spectra of compound **8b**

^13^C NMR spectra of compound **8b**

^1^H NMR spectra of compound **8c**

^13^C NMR spectra of compound **8c**

^1^H NMR spectra of compound **9a**

^13^C NMR spectra of compound **9a**

^1^H NMR spectra of compound **9b**

^13^C NMR spectra of compound **9b**

^1^H NMR spectra of compound **9c**

^13^C NMR spectra of compound **9c**
