# Supplementary material for: Design, Synthesis, and Biological Evaluation of Benzimidazol‐2‐One Hybrids as Potential Anti‐Alzheimer's Disease Agents
Source: Drug Dev Res. 2026 Aug 20;87(6):e70369. doi: 10.1002/ddr.70369 (PMC13492513; doi:10.1002/ddr.70369)
Supplement: Supplementary file 2 [file DDR-87-e70369-s002.docx]

**Suplementary Table 1. Chromatographic Retention Times and Multi‑Wavelength Response Intensities (280, 595, 517, 450 nm) of the Fifteen Synthesized Compounds**

|  | **Retention Time (RT) (min.)** | **Concentrations (for 280 nm) (ppm)** | **Concentrations (for 595 nm) (ppm)** | **Concentrations (for 517 nm) (ppm)** | **Concentrations (for 450 nm) (ppm)** |
| --- | --- | --- | --- | --- | --- |
| **1** | 10.762 | 145.177 ±0.014 | 44.369 ±0.010 | 28.584 ±0.006 | 84.797 ±0.007 |
| **2** | 21.034 | 139.811 ±0.009 | 60.817 ±0.005 | 73.334 ±0.005 | 119.841 ±0.006 |
| **3** | 22.105 | 64.345 ±0.013 | 41.355 ±0.005 | 32.370 ±0.015 | 111.331 ±0.011 |
| **4a** | 8.516 | 46.688 ±0.003 | 63.165 ±0.015 | 102.938 ±0.001 | 76.070 ±0.009 |
| **4b** | 9.744 | 116.767 ±0.011 | 56.323 ±0.004 | 60.752 ±0.015 | 100.828 ±0.009 |
| **4c** | 6.916 | 38.067 ±0.011 | 120.236 ±0.013 | 128.193 ±0.003 | 103.998 ±0.007 |
| **7a** | 16.870 | 58.131 ±0.002 | 24.023 ±0.011 | 124.806 ±0.013 | 103.222 ±0.002 |
| **7b** | 19.738 | 144.736 ±0.007 | 47.642 ±0.003 | 140.192 ±0.005 | 67.003 ±0.008 |
| **7c** | 18.118 | 61.556 ±0.014 | 148.110 ±0.001 | 83.297 ±0.005 | 119.087 ±0.015 |
| **8a** | 11.768 | 65.645 ±0.003 | 149.723 ±0.015 | 20.393 ±0.004 | 120.276 ±0.010 |
| **8b** | 13.061 | 146.431 ±0.012 | 46.541 ±0.003 | 19.384 ±0.006 | 41.393 ±0.014 |
| **8c** | 2.282 | 122.211 ±0.009 | 125.957 ±0.011 | 19.847 ±0.004 | 137.518 ±0.005 |
| **9a** | 4.744 | 116.363 ±0.004 | 114.037 ±0.015 | 45.268 ±0.007 | 147.068 ±0.009 |
| **9b** | 14.318 | 95.046 ±0.008 | 79.587 ±0.011 | 124.085 ±0.010 | 10.460 ±0.005 |
| **9c** | 3.026 | 142.072 ±0.012 | 90.771 ±0.007 | 133.849 ±0.002 | 55.822 ±0.004 |

**Suplementary Table 2. Limit of Detection (LOD) and Limit of Quantitation (LOQ) Values of the Fifteen Synthesized Compounds at Four Wavelengths (280, 595, 517, 450 nm)**

|  | **LOD, ppm, 280 nm** | **LOQ, ppm, 280 nm** | **LOD, ppm, 595 nm** | **LOQ, ppm, 595 nm** | **LOD, ppm, 517 nm** | **LOQ, ppm, 517 nm** | **LOD, ppm, 450 nm** | **LOQ, ppm, 450 nm** |
| --- | --- | --- | --- | --- | --- | --- | --- | --- |
| **1** | 0.174 ±0.009 | 0.574 ±0.006 | 0.066 ±0.009 | 0.218 ±0.002 | 0.075 ±0.007 | 0.247 ±0.012 | 0.021 ±0.009 | 0.069 ±0.010 |
| **2** | 0.040 ±0.012 | 0.132 ±0.011 | 0.136 ±0.003 | 0.449 ±0.014 | 0.145 ±0.006 | 0.478 ±0.014 | 0.185 ±0.014 | 0.610 ±0.001 |
| **3** | 0.147 ±0.013 | 0.485 ±0.007 | 0.152 ±0.005 | 0.502 ±0.003 | 0.134 ±0.011 | 0.442 ±0.011 | 0.174 ±0.009 | 0.574 ±0.010 |
| **4a** | 0.071 ±0.009 | 0.234 ±0.010 | 0.173 ±0.007 | 0.571 ±0.004 | 0.096 ±0.001 | 0.317 ±0.004 | 0.063 ±0.007 | 0.208 ±0.004 |
| **4b** | 0.198 ±0.013 | 0.653 ±0.013 | 0.105 ±0.005 | 0.346 ±0.011 | 0.042 ±0.013 | 0.139 ±0.014 | 0.146 ±0.011 | 0.482 ±0.013 |
| **4c** | 0.145 ±0.007 | 0.478 ±0.012 | 0.062 ±0.003 | 0.205 ±0.004 | 0.171 ±0.006 | 0.564 ±0.012 | 0.192 ±0.011 | 0.634 ±0.003 |
| **7a** | 0.166 ±0.002 | 0.548 ±0.007 | 0.057 ±0.003 | 0.188 ±0.001 | 0.077 ±0.010 | 0.254 ±0.008 | 0.188 ±0.007 | 0.620 ±0.008 |
| **7b** | 0.063 ±0.009 | 0.208 ±0.001 | 0.084 ±0.012 | 0.277 ±0.011 | 0.106 ±0.013 | 0.350 ±0.004 | 0.145 ±0.014 | 0.478 ±0.012 |
| **7c** | 0.107 ±0.004 | 0.353 ±0.002 | 0.155 ±0.006 | 0.511 ±0.006 | 0.123 ±0.006 | 0.406 ±0.012 | 0.037 ±0.012 | 0.122 ±0.005 |
| **8a** | 0.172 ±0.002 | 0.568 ±0.014 | 0.184 ±0.009 | 0.607 ±0.003 | 0.063 ±0.010 | 0.208 ±0.002 | 0.128 ±0.006 | 0.422 ±0.009 |
| **8b** | 0.062 ±0.014 | 0.205 ±0.006 | 0.026 ±0.009 | 0.086 ±0.010 | 0.062 ±0.003 | 0.205 ±0.013 | 0.172 ±0.014 | 0.568 ±0.014 |
| **8c** | 0.161 ±0.012 | 0.531 ±0.005 | 0.039 ±0.007 | 0.129 ±0.014 | 0.084 ±0.014 | 0.277 ±0.005 | 0.121 ±0.004 | 0.399 ±0.010 |
| **9a** | 0.074 ±0.009 | 0.244 ±0.007 | 0.111 ±0.009 | 0.366 ±0.006 | 0.028 ±0.011 | 0.092 ±0.002 | 0.032 ±0.010 | 0.106 ±0.002 |
| **9b** | 0.084 ±0.011 | 0.277 ±0.003 | 0.073 ±0.003 | 0.241 ±0.004 | 0.064 ±0.006 | 0.211 ±0.011 | 0.156 ±0.009 | 0.515 ±0.002 |
| **9c** | 0.193 ±0.011 | 0.637 ±0.004 | 0.036 ±0.002 | 0.119 ±0.014 | 0.034 ±0.009 | 0.112 ±0.006 | 0.050 ±0.005 | 0.165 ±0.006 |

**Suplementary Table 3. Validation Parameters of the Fifteen Synthesized Compounds**

|  | **Matrix Effect (%)** | **Interday Precision (%)** | **Intraday Precision (%)** | **Recovery (%)** | **Repeatability (%)** | **R^²^** | **Calibration Curve Equation** |
| --- | --- | --- | --- | --- | --- | --- | --- |
| **1** | 99.3 | 2.39 | 3.52 | 99.6 | 104.86 | 0.994 | y = 1.059x + 0.049 |
| **2** | 101.61 | 3.34 | 2.2 | 98.48 | 97.43 | 0.995 | y = 0.881x + 0.024 |
| **3** | 104.45 | 4.22 | 3.26 | 104.77 | 98.17 | 0.996 | y = 0.931x + 0.05 |
| **4a** | 103.99 | 3.22 | 2.19 | 103.0 | 98.94 | 0.994 | y = 0.983x + 0.014 |
| **4b** | 95.71 | 4.5 | 1.34 | 100.69 | 104.21 | 0.992 | y = 0.886x + 0.049 |
| **4c** | 104.2 | 4.96 | 4.52 | 104.7 | 101.85 | 0.995 | y = 0.937x + 0.037 |
| **7a** | 97.93 | 1.12 | 2.63 | 95.28 | 98.06 | 0.999 | y = 0.874x + 0.006 |
| **7b** | 100.76 | 3.79 | 4.21 | 100.33 | 95.72 | 0.992 | y = 0.936x + 0.039 |
| **7c** | 96.09 | 3.4 | 4.17 | 97.05 | 97.25 | 0.991 | y = 1.103x + 0.042 |
| **8a** | 95.71 | 2.28 | 1.3 | 99.95 | 100.87 | 0.996 | y = 1.018x + 0.037 |
| **8b** | 102.0 | 3.51 | 3.96 | 98.95 | 101.12 | 0.991 | y = 0.987x + 0.009 |
| **8c** | 99.09 | 4.42 | 4.41 | 102.21 | 103.01 | 0.997 | y = 0.878x + 0.017 |
| **9a** | 99.78 | 4.49 | 1.38 | 95.59 | 100.57 | 0.998 | y = 0.942x + 0.035 |
| **9b** | 103.36 | 4.77 | 2.02 | 100.35 | 101.98 | 0.992 | y = 0.935x + 0.049 |
| **9c** | 100.65 | 1.38 | 4.45 | 99.73 | 96.69 | 0.998 | y = 1.123x + 0.041 |

**Suplementary Table 4. Chromatographic and Multi‑Wavelength Detection Characteristics of the Liposomal Forms of Fifteen Hybrid Molecules**

|  | **Retention Time (RT) (min.)** | **Concentrations (for 280 nm) (ppm)** | **Concentrations (for 595 nm) (ppm)** | **Concentrations (for 517 nm) (ppm)** | **Concentrations (for 450 nm) (ppm)** |
| --- | --- | --- | --- | --- | --- |
| **1** | 12.0 | 130.01 ±0.011 | 138.685 ±0.012 | 64.152 ±0.003 | 32.732 ±0.003 |
| **2** | 22.0 | 107.817 ±0.005 | 55.134 ±0.014 | 56.699 ±0.003 | 103.015 ±0.014 |
| **3** | 23.25 | 125.785 ±0.007 | 148.853 ±0.001 | 63.986 ±0.002 | 25.96 ±0.011 |
| **4a** | 9.5 | 23.937 ±0.007 | 48.207 ±0.006 | 17.774 ±0.009 | 13.629 ±0.002 |
| **4b** | 10.75 | 149.623 ±0.011 | 134.397 ±0.003 | 72.626 ±0.003 | 17.79 ±0.014 |
| **4c** | 8.25 | 135.32 ±0.011 | 149.318 ±0.008 | 137.771 ±0.012 | 53.93 ±0.008 |
| **7a** | 18.25 | 45.744 ±0.004 | 20.228 ±0.015 | 28.441 ±0.003 | 129.676 ±0.007 |
| **7b** | 20.75 | 28.185 ±0.003 | 146.559 ±0.008 | 110.552 ±0.002 | 103.931 ±0.002 |
| **7c** | 19.5 | 41.726 ±0.005 | 80.284 ±0.001 | 132.723 ±0.014 | 12.192 ±0.013 |
| **8a** | 13.25 | 51.791 ±0.006 | 113.643 ±0.01 | 127.98 ±0.008 | 147.413 ±0.004 |
| **8b** | 14.5 | 52.361 ±0.006 | 79.047 ±0.011 | 110.282 ±0.013 | 76.43 ±0.006 |
| **8c** | 3.25 | 122.683 ±0.014 | 121.741 ±0.01 | 137.689 ±0.006 | 30.407 ±0.014 |
| **9a** | 5.75 | 28.43 ±0.007 | 16.03 ±0.005 | 85.512 ±0.012 | 87.399 ±0.007 |
| **9b** | 15.75 | 85.781 ±0.011 | 117.71 ±0.011 | 75.813 ±0.012 | 141.098 ±0.007 |
| **9c** | 4.5 | 81.379 ±0.01 | 52.639 ±0.008 | 28.368 ±0.012 | 15.949 ±0.008 |

**Suplementary Table 5. Analytical Sensitivity Parameters (LOD and LOQ) of the Liposomal Formulations of Fifteen Synthesized Compounds at 280, 595, 517, and 450 nm**

|  | **LOD, ppm, 280 nm** | **LOQ, ppm, 280 nm** | **LOD, ppm, 595 nm** | **LOQ, ppm, 595 nm** | **LOD, ppm, 517 nm** | **LOQ, ppm, 517 nm** | **LOD, ppm, 450 nm** | **LOQ, ppm, 450 nm** |
| --- | --- | --- | --- | --- | --- | --- | --- | --- |
| **1** | 0.143 ±0.015 | 0.472 ±0.008 | 0.055 ±0.014 | 0.181 ±0.003 | 0.087 ±0.003 | 0.287 ±0.003 | 0.041 ±0.011 | 0.135 ±0.004 |
| **2** | 0.04 ±0.014 | 0.132 ±0.009 | 0.051 ±0.002 | 0.168 ±0.012 | 0.096 ±0.015 | 0.317 ±0.013 | 0.187 ±0.009 | 0.617 ±0.002 |
| **3** | 0.117 ±0.013 | 0.386 ±0.011 | 0.189 ±0.013 | 0.624 ±0.009 | 0.091 ±0.008 | 0.3 ±0.013 | 0.121 ±0.006 | 0.399 ±0.003 |
| **4a** | 0.154 ±0.014 | 0.508 ±0.013 | 0.057 ±0.013 | 0.188 ±0.011 | 0.139 ±0.004 | 0.459 ±0.01 | 0.166 ±0.006 | 0.548 ±0.001 |
| **4b** | 0.154 ±0.011 | 0.508 ±0.005 | 0.193 ±0.011 | 0.637 ±0.001 | 0.185 ±0.006 | 0.61 ±0.01 | 0.048 ±0.013 | 0.158 ±0.014 |
| **4c** | 0.038 ±0.007 | 0.125 ±0.01 | 0.162 ±0.008 | 0.535 ±0.002 | 0.069 ±0.014 | 0.228 ±0.008 | 0.06 ±0.001 | 0.198 ±0.007 |
| **7a** | 0.053 ±0.014 | 0.175 ±0.008 | 0.166 ±0.004 | 0.548 ±0.014 | 0.098 ±0.004 | 0.323 ±0.005 | 0.071 ±0.003 | 0.234 ±0.007 |
| **7b** | 0.067 ±0.012 | 0.221 ±0.003 | 0.176 ±0.008 | 0.581 ±0.015 | 0.097 ±0.001 | 0.32 ±0.008 | 0.167 ±0.006 | 0.551 ±0.013 |
| **7c** | 0.135 ±0.008 | 0.446 ±0.012 | 0.142 ±0.008 | 0.469 ±0.012 | 0.111 ±0.004 | 0.366 ±0.009 | 0.129 ±0.011 | 0.426 ±0.013 |
| **8a** | 0.053 ±0.007 | 0.175 ±0.006 | 0.159 ±0.012 | 0.525 ±0.002 | 0.107 ±0.003 | 0.353 ±0.006 | 0.135 ±0.006 | 0.446 ±0.012 |
| **8b** | 0.192 ±0.005 | 0.634 ±0.008 | 0.194 ±0.012 | 0.64 ±0.001 | 0.1 ±0.01 | 0.33 ±0.012 | 0.166 ±0.008 | 0.548 ±0.006 |
| **8c** | 0.164 ±0.003 | 0.541 ±0.013 | 0.086 ±0.004 | 0.284 ±0.013 | 0.177 ±0.002 | 0.584 ±0.015 | 0.132 ±0.005 | 0.436 ±0.003 |
| **9a** | 0.057 ±0.004 | 0.188 ±0.007 | 0.16 ±0.008 | 0.528 ±0.015 | 0.05 ±0.003 | 0.165 ±0.004 | 0.051 ±0.006 | 0.168 ±0.014 |
| **9b** | 0.077 ±0.002 | 0.254 ±0.01 | 0.073 ±0.007 | 0.241 ±0.011 | 0.183 ±0.012 | 0.604 ±0.003 | 0.116 ±0.008 | 0.383 ±0.013 |
| **9c** | 0.184 ±0.002 | 0.607 ±0.008 | 0.103 ±0.01 | 0.34 ±0.013 | 0.15 ±0.01 | 0.495 ±0.01 | 0.191 ±0.007 | 0.63 ±0.01 |

**Suplementary Table 6. Validation Metrics for the Liposomal Forms of Fifteen Hybrid Compounds**

|  | **Specificity (%)** | **Matrix Effect (%)** | **Interday Precision (%)** | **Intraday Precision (%)** | **Recovery (%)** | **Repeatability (%)** | **R²** | **Calibration Curve Equation** |
| --- | --- | --- | --- | --- | --- | --- | --- | --- |
| **1** | 96.04 | 97.7 | 2.18 | 3.39 | 101.25 | 101.61 | 0.991 | y = 0.954x + 0.023 |
| **2** | 101.5 | 98.51 | 2.7 | 4.47 | 95.6 | 101.07 | 0.998 | y = 1.116x + 0.045 |
| **3** | 104.58 | 96.82 | 1.0 | 4.38 | 99.04 | 98.39 | 0.998 | y = 1.132x + 0.02 |
| **4a** | 95.32 | 100.7 | 4.53 | 2.11 | 100.88 | 100.86 | 0.994 | y = 0.892x + 0.006 |
| **4b** | 99.23 | 99.23 | 3.14 | 1.54 | 101.51 | 99.69 | 0.995 | y = 1.147x + 0.01 |
| **4c** | 98.21 | 95.24 | 2.57 | 2.16 | 104.1 | 100.01 | 0.997 | y = 1.132x + 0.011 |
| **7a** | 104.97 | 104.05 | 4.1 | 4.47 | 102.23 | 97.54 | 0.994 | y = 0.926x + 0.013 |
| **7c** | 95.37 | 100.75 | 2.5 | 4.52 | 99.88 | 102.52 | 0.992 | y = 0.998x + 0.016 |
| **7b** | 101.87 | 104.69 | 2.65 | 4.61 | 98.33 | 98.83 | 0.998 | y = 1.141x + 0.017 |
| **8a** | 102.45 | 104.95 | 3.14 | 1.83 | 102.68 | 101.22 | 0.99 | y = 1.012x + 0.047 |
| **8b** | 104.49 | 100.35 | 1.28 | 4.56 | 100.3 | 98.88 | 0.998 | y = 0.995x + 0.001 |
| **8c** | 97.3 | 98.63 | 3.24 | 1.41 | 98.44 | 103.16 | 0.991 | y = 0.886x + 0.012 |
| **9a** | 99.22 | 104.03 | 4.36 | 1.06 | 100.67 | 102.12 | 0.992 | y = 1.147x + 0.02 |
| **9b** | 100.86 | 98.51 | 1.72 | 1.91 | 99.86 | 99.94 | 0.994 | y = 1.127x + 0.045 |
| **9c** | 101.92 | 104.87 | 4.56 | 2.05 | 103.04 | 95.06 | 0.993 | y = 0.94x + 0.019 |

**Suplementary Table 7.** Predicted physicochemical, pharmacokinetic, and drug-likeness properties of the synthesized compounds and reference drugs (SwissADME).

| **Compound** | **MW (g/mol)** | **TPSA (Å²)** | **cLogP*** | **GI abs.** | **BBB** | **P-gp sub.** | **Lipinski (viol.)** | **Bioavail.** | **Brenk** |
| --- | --- | --- | --- | --- | --- | --- | --- | --- | --- |
| **1** | 162.19 | 48.65 | 1.63 | High | Yes | No | 0 | 0.55 | 0 |
| **2** | 334.37 | 79.53 | 0.94 | High | No | No | 0 | 0.55 | 1 |
| **3** | 306.32 | 137.17 | −2.10 | Low | No | No | 0 | 0.55 | 2 |
| **4a** | 518.74 | 39.67 | 2.51 | High | Yes | No | 1 | 0.55 | 0 |
| **4b** | 587.63 | 39.67 | 3.80 | High | Yes | No | 2 | 0.17 | 0 |
| **4c** | 554.72 | 39.67 | 3.33 | High | Yes | No | 1 | 0.55 | 0 |
| **7a** | 650.59 | 203.75 | 0.17 | Low | No | Yes | 2 | 0.17 | 1 |
| **7b** | 719.48 | 203.75 | 1.29 | Low | No | Yes | 2 | 0.17 | 1 |
| **7c** | 808.39 | 203.75 | 1.44 | Low | No | No | 2 | 0.17 | 1 |
| **8a** | 666.69 | 289.37 | 0.10 | Low | No | Yes | 3 | 0.17 | 3 |
| **8b** | 576.69 | 197.43 | 1.65 | Low | No | No | 3 | 0.17 | 1 |
| **8c** | 638.63 | 289.37 | −0.61 | Low | No | Yes | 3 | 0.17 | 3 |
| **9a** | 444.58 | 165.95 | 0.32 | Low | No | Yes | 0 | 0.55 | 1 |
| **9b** | 540.66 | 165.95 | 2.25 | Low | No | Yes | 1 | 0.55 | 1 |
| **9c** | 630.66 | 257.59 | 0.66 | Low | No | Yes | 2 | 0.17 | 3 |
| Donepezil | 379.49 | 38.77 | 3.15 | High | Yes | Yes | 0 | 0.55 | 0 |
| Selegiline | 187.28 | 3.24 | 2.42 | High | Yes | No | 0 | 0.55 | 1 |

* cLogP corresponds to the SwissADME Consensus Log P_o/w_ value. GI abs., gastrointestinal absorption; BBB, blood–brain barrier permeation; P-gp sub., P-glycoprotein substrate; Bioavail., Abbott bioavailability score. All compounds returned zero PAINS alerts.
